# Identification and functional analysis of the ICK gene family in maize

**DOI:** 10.1038/srep43818

**Published:** 2017-03-06

**Authors:** Qianlin Xiao, Chunxia Zhang, Hui Li, Bin Wei, Yongbin Wang, Huanhuan Huang, Yangping Li, Guowu Yu, Hanmei Liu, Junjie Zhang, Yinghong Liu, Yufeng Hu, Yubi Huang

**Affiliations:** 1 College of Agronomy, Sichuan Agricultural University, Chengdu 611130, Sichuan, China; 2 College of Life Science, Sichuan Agricultural University, Ya’an 625014, Sichuan, China; 3 Maize Research Institute, Sichuan Agricultural University, Chengdu 611130, Sichuan, China

## Abstract

Inhibitors of cyclin-dependent kinases (ICKs) are key regulators of cyclin-dependent kinase activities and cell division. Herein, we identified eight ICKs in maize, which we named Zeama;ICKs (ZmICKs). Primary sequencing and phylogenetic analyses were used to divide the ZmICK family into two classes: group B and group C. Subcellular localization analysis of ZmICK:enhanced green fluorescent protein (eGFP) fusion constructs in tobacco leaf cells indicated that ZmICKs are principally nuclear. Co-localization analysis of the ZmICKs and maize A-type cyclin-dependent kinase (ZmCDKA) was also performed using enhanced green fluorescent protein (eGFP) and red fluorescent protein (RFP) fusion constructs. The ZmICKs and ZmCDKA co-localized in the nucleus. Semi-quantitative RT-PCR analysis of the ZmICKs showed that they were expressed at different levels in all tissues examined and shared similar expression patterns with cell cycle-related genes. Yeast two-hybrid and bimolecular fluorescence complementation assays showed that ZmICK1, ZmICK2, ZmICK3, and ZmICK4 interact with ZmCDKA1 and ZmCDKA3. Interestingly, ZmICK7 interacts with D-type cyclins. Transformed and expressed ZmCDKA1 and ZmICKs together in fission yeast revealed that ZmICK1, ZmICK3, and ZmICK4 can affect ZmCDKA1 function. Moreover, the C-group of ZmICKs could interact with ZmCDKA1 directly and affect ZmCDKA1 function, suggesting that C-group ZmICKs are important for cell division regulation.

The development of plant organs is directly dependent on the frequency of cell division, the parameters of the cell cycle, and the number and size of the cells. To control the cell cycle and cell division, plants must modulate the activity of cyclin-dependent kinases (CDKs) during development and coordinate this activity with nutritional, hormonal, and environmental signals. The activity of CDKs is regulated by several biochemical mechanisms, including phosphorylation and dephosphorylation, direct binding by cyclins and cyclin-dependent kinase inhibitors (inhibitors of cyclin-dependent kinase [ICK] or Kip-related proteins [KRP]), and other signalling proteins that participate in the regulation of cell division^1^.

The ICK/KRP proteins directly interact with cyclins, CDKs, or cyclin-CDK complexes and regulate the activity of cyclin-dependent kinases^2^. The first plant cyclin-dependent kinase inhibitor was cloned from *Arabidopsis thaliana* using a yeast two-hybrid system, which verified the interaction between ICK/KRP proteins and CDKs^3^. ICK/KRP family proteins have been identified in several plants. Seven ICK/KRP genes have been identified in *Arabidopsis*^3^,^4^,^5^,^6^, seven *ICK/KRP* genes (including one pseudogene) have been identified in *Oryza sativa*^7^,^8^,^9^, and two *ICK/KRP* genes have been identified in maize endosperm^10^. *ICK/KRP* genes have also been found in tomato, tobacco, and alfalfa^11^,^12^,^13^.

Plant ICK/KRP proteins harbour a characteristic conserved short C-terminal region^4^,^5^, whereas the other regions show sequence divergence among the different ICK/KRP proteins from various types of plants, which can be indicative of different functions among the members. Subcellular localization studies of the ICK/KRP proteins based on multiple sequence analyses and fusion to green fluorescent protein have indicated that all seven *Arabidopsis* ICK/KRPs are located in the nucleus^14^,^15^,^16^,^17^. Moreover, two rice ICK/KRPs have also been shown to localize to the nucleus^9^, suggesting that the nucleus is the main functional cellular compartment for plant CDK inhibitors. A deletion study of ICK/KRP proteins in *Arabidopsis* identified several nuclear localization signals (NLS), including YLQLRSRRL^14^,^18^, in four *Arabidopsis* ICKs. The conserved YLQLRSRRL motif is also present in four of the rice ICK/KRP proteins^9^.

Previous studies have shown that the ICK/KRP proteins interact with the A-type CDKs through the conserved C-terminal region^5^,^19^,^20^. In *Arabidopsis*, ICK1 can interact with CDKA;1, CycD1, CycD2, and CycD3; deletion analysis indicated that the conserved C-terminal region of ICK1 is important for interaction with CDKA;1, whereas a region upstream of the conserved C-terminus is important for interaction with D-type cyclins^5^,^20^. ICK1 lacking the N-terminal region strongly interacts with *Arabidopsis* CDKA;1, which suggests that the N-terminus of ICK1 may negatively regulate this interaction. Additionally, the N-terminus can interact with CYCD3;1^15^. In rice, OsiICK1 and OsiICK6 interact with D-type cyclins, but they differ in their interactions with Orysa;CDKA;1^9^. Orysa;KRP3 can bind Orysa;CDKA;1, Orysa;CDKA;2, Orysa;CycA1;1, and Orysa;CycD2;2^21^. In maize, tomato, tobacco, and alfalfa, ICKs can also associate with different proteins, such as D-type cyclins, A-type cyclins, and A-type CDKs^10^,^11^,^13^,^22^,^23^.

Research on different plants has contributed to our understanding of plant ICK function. Previous studies have shown that plants over-expressing ICK/KRP genes display some common phenotypes, such as reduced plant size and serrated leaves, with fewer but larger cells^2^,^4^,^7^,^12^,^22^,^24^,^25^. Moreover, the down-regulation of multiple ICK/KRP genes also affects organ development and seed size^26^. ICKs are also associated with the formation of tissues and organs^27^. At the cellular level, ICK transgenic plants display reduced ploidy due to the inhibition of endoreduplication^4^,^7^,^12^,^22^,^28^. However, the slight over-expression of *ICK2* in *Arabidopsis* caused cells to enter endoreduplication earlier and display higher ploidy^29^. In rice, the over-expression of *OsiICK6* also led to a variety of phenotypes, including changes in plant growth, morphology, pollen viability, and seed setting^9^. *Orysa;KRP1* is associated with endosperm development and seed filling in rice, whereas *Orysa;KRP3* plays an important role in the development of the syncytial endosperm^7^,^21^. Studies in tobacco and alfalfa have shown similar results to *Arabidopsis* and rice^13^,^19^. Furthermore, studies have indicated that ICK1 can move between cells, suggesting that ICK1 can affect multiple cells^30^.

The expression of the ICK/KRP genes varies during the cell cycle^31^ and is tissue-specific^4^,^5^,^13^,^19^,^20^,^32^. Several ICK/KRP genes are affected by environmental signals, such as salt levels and hormones^13^,^20^. Although the ICK/KRP genes have been investigated in various plants^33^, studies in maize remain scarce. The maize genome was completely sequenced in 2009^34^, providing an important tool for the identification and analysis of all of the genes in the genome.

In this article, we report the identification of nine *ZmICK* genes in the maize genome; these genes encode 8 proteins, each containing a cyclin-dependent kinase inhibitor (CDI) region, and two of the identified genes possess similar gene structure, cDNA sequence, and protein sequence. Primary sequence analyses of the putative ICK/KRP proteins in maize indicated that the maize genes identified share a conserved C-terminal region and possess a gene structure similar to those of the ICK/KRP genes of other plants. However, we were unable to clone one of the genes identified in the reference genome as containing the CDI region. Based on the primary sequence analysis and functional studies, we determined that the 8 candidate genes in maize endosperm, which include two genes described by Coelho *et al*.^10^, are ICK/KRP genes.

## Results

### Identification and preliminary analysis of putative ICK family genes in maize

Inhibitors of cyclin-dependent kinase (ICKs) have a crucial role in regulating the activity of cyclin-dependent kinases and endoreduplication. To search for novel maize ICK family members, the maize proteome was screened for proteins containing the highly conserved CDI^35^ region. We identified 8 candidate proteins encoded by 9 gene loci as maize ICKs; these proteins will hereafter be referred to as Zeama;ICK;1 (ZmICK1) to Zeama;ICK;8 (ZmICK8) (Table S1). Notably, the GRMZM2G084570 and GRMZM5G854731 loci display the same sequence, encode the same protein, and are thus hereafter referred to as *ZmICK6.1* and *ZmICK6.2*. Subsequent experiments determined that these two loci are the same gene, *ZmICK6*. As shown in Figure S1, the nine putative ZmICK genes were found on chromosomes 1, 4, 5, and 9; only *ZmICK6.2* was located on chromosome 8. The basic information for the candidate genes and the gene IDs corresponding to the gene names are provided in Table S1.

### Conserved motif and gene structure analysis and evolutionary tree construction of the *ZmICK* genes

*ICK/KRP* genes have been identified in several plants through sequence alignment and functional analysis. To classify the 9 *ICK/KRP* genes identified in this study, 31 other *ICK/KRP* genes from five species (*Arabidopsis thaliana* [7 *ICK/KRP* genes], *Oryza sativa* [6 *ICK/KRP* genes], *Brachypodium distachyon* [7 *ICK/KRP* genes], *Glycine max* [5 *ICK/KRP* genes], and *Populus trichocarpa* [6 *ICK/KRP* genes]) were obtained, and neighbour-joining phylogenetic trees were constructed as reported by Torres-Acosta^33^. The *ICK/KRP* genes could be divided into three groups based on the number of cotyledons in the plants. Groups A and B only contained proteins from the dicotyledonous or monocotyledonous plants, respectively. Group C contained *ICK/KRP* genes from both dicotyledons and monocotyledons. With regard to the 8 *ZmICK* genes identified in this study, *ZmICK5* to *ZmICK8* belong to group B, and *ZmICK1* to *ZmICK4* belong to group C (Figure S2). *ZmICK1* and *ZmICK5* were previously reported by Coelho *et al*.^10^ in maize endosperm, where they were named *ZmKRP1* and *ZmKRP2*. However, this research screened all the ICK/KRP genes in maize genome and phylogenetic analysis indicates maize ICK gene divided into two groups. *ZmKRP1* and *ZmKRP2* belong to different *ICK/KRP* groups, and we renamed these genes for our study. Conserved motif and gene structure analysis also verified the classification of the phylogenetic analysis. Forty ICK/KRP proteins were assessed using the MEME analysis tool, and a total of 10 conserved motifs were identified. Motif 1 and motif 3 were identified as comprising the conserved C-terminus. Motif 4 is the conserved N-terminal domain, which is important for interactions with D-type cyclins^15^. Motif 2 contains a sequence related to the nuclear localization signal (NLS)^14^,^18^. The functions of the remaining motifs remain unclear. Most members of group A only contain motifs 1 and 3, which comprise the conserved C-terminus that is important for the function of the ICK/KRP proteins. The remaining two ICK protein groups also harbour specific motifs in addition to motifs 1 and 3 that contribute to their functions. The sequences of the conserved maize motifs are shown in Table S4.

Gene structure analysis indicated that the *ICK/KRP* genes of the six species share a similar structure. With the exception of *ZmICK8*, which has only 2 exons, the class A and class B genes consist of 3 or 4 exons. Compared with class C, class A and class B consist of smaller genes. With the exception of *AtICK3* of *Arabidopsis* and *PtKRP2* of *Populus trichocarpa*, both of which contain a small intron, the class C ICK/KRP genes consist of 3 exons and a large intron.

**Figure 1:**
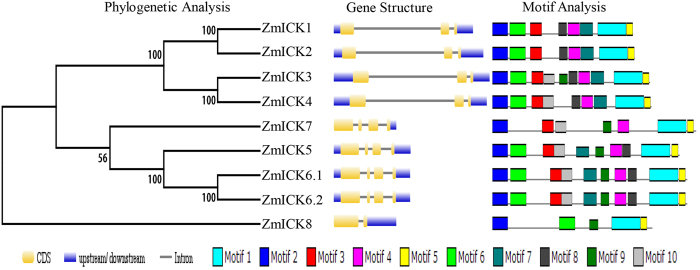
The primary sequence analysis of the maize ICK gene family. Evolutionary trees were constructed using MEGA 5.10 software, the number on the branches represent the bootstrap. The three main components in gene structure analysis represent CDS region, upstream/downstream and Intron, respectively. The different color boxes represent the different motif through MEME analysis.

The results of the phylogenetic, conserved motif and gene structure analyses of the maize ICK/KRP genes are shown in Fig. 1. ZmICK1, ZmICK2, ZmICK3, and ZmICK4 cluster in a branch of the evolutionary tree as group C. However, group B contains four ZmICK proteins that do not cluster to the same branch in the evolutionary tree. ZmICK5, ZmICK6, and ZmICK7 are closely related, whereas ZmICK8 is unique compared with the other seven ZmICK genes. ZmICK8 also has unique characteristics in terms of genetic structure and conserved motifs.

### Gene cloning and protein sequence analysis of *ZmICKs*

To further study the function of the *ZmICK* genes, *ZmICK* genes were cloned from the maize inbred line 18-599, and further experiments were performed. The *ZmICK1* to *ZmICK6* sequences from inbred line 18-599 were identical those of the B73 reference sequence, but differences were found in the cloned and reference sequences of *ZmICK7* and *ZmICK8*. The cloned *ZmICK7* sequence included three inserted bases, which encoded the amino acid insertion shown in Figure S3B. The cloned *ZmICK8* contained an insertion element, which results in early termination of translation and loss of the conserved CDI region. We cloned the *ZmICK8* genes from three other inbred lines and found the same early termination insertion. The alignment of the cloned *ZmICK8* sequence and the reference sequence is shown in Figure S3C; *ZmICK8* was not included in the following experiments.

Motif analyses of the cloned sequences indicate that both classes of ZmICK, aside from ZmICK8, contain motifs 1 and 3, which comprises the CDI region. Motifs 2, 4, and 6 were also present in the two groups. Group C contained the specific motifs 5, 7, and 8. Group B contained motifs 9 and 10, as shown in Figure S3D.

### Expression pattern analysis of the ZmICK genes

**Figure 2:**
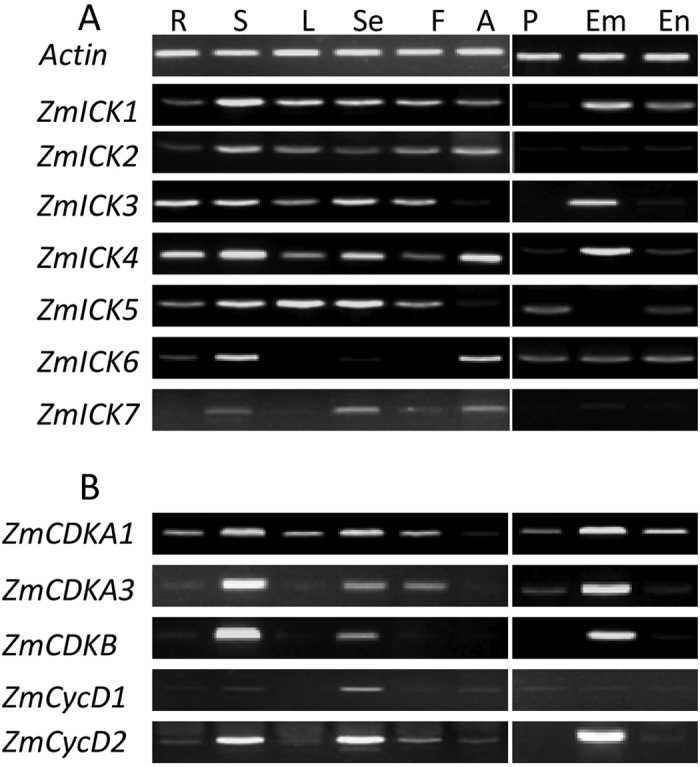
(**A**) Semi-quantitative RT-PCR analysis of the putative maize ICK/KRP genes in different maize tissues. (**B**) Semi-quantitative RT-PCR analysis of five maize cell cycle-related genes. R represents the root; S is the stem; L indicates the leaf; F is the filament; A indicates the anther; and Se, Em, En, P represent the seed 10 days after pollination, the embryo, the endosperm and the pericarp of the seed 15 days after pollination, respectively. *Actin* is the internal reference gene.

Expression pattern analysis is the foundation of any gene function study, and expression pattern analysis of the seven *ZmICK* genes in different tissues indicated that the *ZmICK* genes were expressed at different levels in all of the tissues analysed. As shown in Fig. 2, semi-quantitative PCR analysis revealed that expression of *ZmICK1, ZmICK2, ZmICK3*, and *ZmICK4* was the highest in the stem compared with the other tissues. We also observed relatively high *ZmICK1, ZmICK3, ZmICK4*, and *ZmICK5* expression in the seed 10 days after pollination (DAP), but *ZmICK6* was expressed at higher levels in the leaf, stem, and anther than in the seed. *ZmICK* expression was lower in the endosperm and seed coat than in the embryo. These results indicate that the expression of *ZmICKs* is higher in young and tender tissues than in mature tissues. Semi-quantitative analysis of four cell cycle-related genes also showed that these genes are highly expressed in the tender tissues. Sequence alignment analysis indicates that *ZmCDKA1* and *ZmCDKA2* share a high level of similarity, and these proteins could not be distinguished in our experiments. The alignment results are shown in Figure S4. ZmCDKA3 is new member of A-type CDK and function remains unclear. Both of them will be served as important subjects in this article^36^.

### Cellular location analysis of ZmICKs and co-localization analysis of group C ZmICKs with ZmCDKA1 and ZmCDKA3

**Figure 3:**
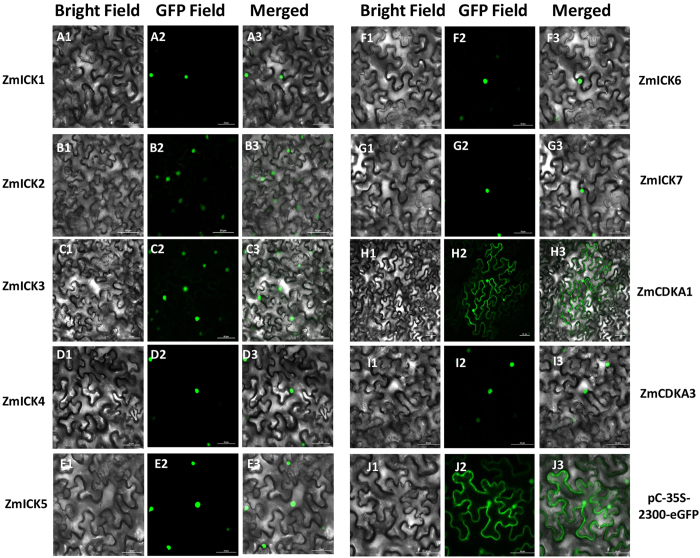
The subcellular localization of the ZmICK proteins and A-type CDK proteins were determined by transforming the ZmICK-eGFP and ZmCDKA-eGFP fusion protein vector into the *Agrobacterium tumefaciens* strain EHA105 and used to infect *Nicotiana benthamiana* leaf cell. (A1-J1) show the tobacco cells under bright-field, (A2-J2) show the location about the fusion protein of ZmICK-eGFP and ZmCDKA-eGFP under the GFP-field, and (A3-J3) is the merged images about the picture of bright-field and GFP-field. Each alphabet labelled with 1 to 3 represents the same gene under the different field. (J1–J3) indicate the localization of the control pCAMBIA2300-35S-eGFP. Bar = 50 μm.

To analyse the subcellular localization of the ZmICK protein family, transit peptide prediction was performed for ZmICK family members. The results of this analysis indicated that the Group B ZmICKs, ZmICK5, ZmICK6, and ZmICK7, contain a putative NLS, indicating that the Group B ZmICKs might localize to the nucleus. No specific NLS was found in the Group C ZmICKs (ZmICK1 to ZmICK4). However, the conserved sequence YLELRSRRL was observed in motif 2 of the Group C proteins, which is similar to the YLQLRSRRL sequence that has been defined as a nuclear localization signal^14^,^18^. Further analysis of the sub-cellular localization of the proteins was conducted by constructing proteins fused with enhanced green fluorescent protein eGFP transforming the expression plasmid into *Agrobacterium* EHA105, and inoculating a *Nicotiana benthamiana* leaf with the transformed Agrobacterium. The results of the sub-cellular localization study of the ZmICKs and ZmCDKAs are shown in Fig. 3. All of the ZmICKs mainly localized to the nucleus. ZmCDKA3 also localized to the nucleus, but ZmCDKA1-eGFP was expressed in both the nucleus and cytoplasm in Fig. 3 shown similar sub-cellular localization with the CDKA1 of *Arabidopsis*^15^.

The co-localization of the Group C ZmICKs and the A-type CDKs was determined by transiently co-expressing the fusion constructs containing ZmICK with enhanced green fluorescent protein (eGFP) and ZmCDKA with red fluorescence protein (RFP) in onion epidermal cells. The results shown in Figure S5 indicate that four Group C ZmICKs and two A-type CDKs share location in the nucleus. It is further to confirm that Group C ZmICKs and A-type CDKs display common localization in the nucleus.

### Interaction analysis of the ZmICKs with cell cycle-related proteins

**Figure 4:**
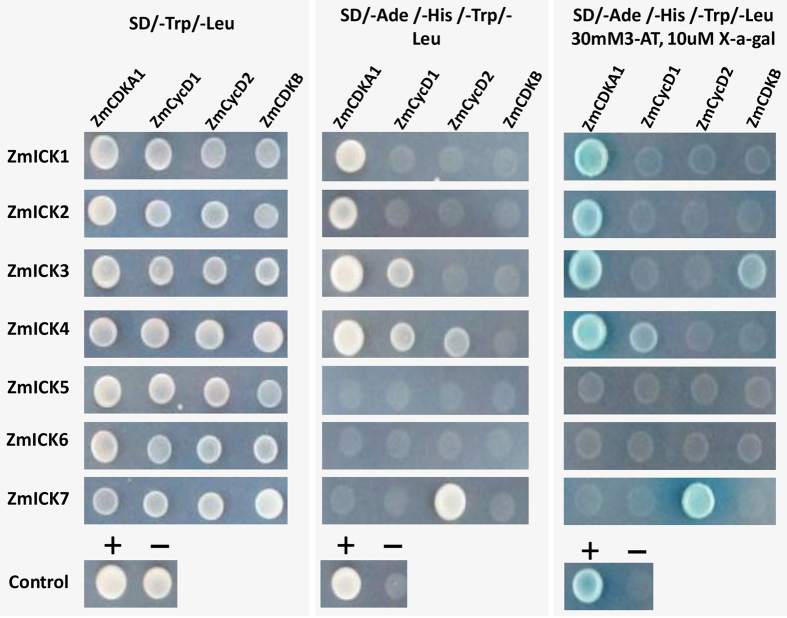
The results of the yeast two-hybrid analysis of ZmICKs and ZmCDKA1, ZmCDKB, ZmCycD1 and ZmCycD2. SD/-Leu/-Trp indicates media lacking tryptophan (Trp) and leucine (Leu). SD/-Ade/-His/-Leu/-Trp represents the synthetic dextrose (SD) media lacking tryptophan (Trp), leucine (Leu), adenine (Ade) and histidine (His). SD/-Ade/-His/-Leu/-Trp/30mM3-AT/10uM X-α-gal indicates the synthetic dextrose (SD) media contain 30 mM 3-AT and 10 uM X-α-gal. The gray box shows the growth condition on the same medium and every yeast plaque indicates the growth of the transformants. “**+**” represents the positive control of the yeast two-hybrid, the combination of pGADT7 and pGBKT7-53. “**−**” Shows the negative control of yeast two-hybrid, the pGADT7-T co-transformed with pGBKT7-lam.

The interactions between ZmICKs and cell cycle-related proteins were evaluated using yeast two-hybrid and bimolecular fluorescence complementation analysis. The yeast two-hybrid results for ZmICKs and the cell cycle proteins are shown in Fig. 4. Yeast cells containing all combinations of ZmICKs and cell cycle proteins grew normally on synthetic dextrose (SD) medium lacking tryptophan (Trp) and leucine (Leu). The yeast expressing the Group C ZmICKs with ZmCDKA1 and ZmICK7 with CycD2 not only grew normally on SD/-Ade/-Leu/-Trp/-His media but also grew normally on SD/-Ade/-Leu/-Trp/-His medium supplemented with 30 mM 3-AT and 10 μl X-α-gal. These strains also had the ability to activate the expression of MET1 to hydrolyse X-α-gal and developed blue colonies. Yeast co-expressing ZmICK3 with ZmCDKB and ZmICK4 with ZmCycD1 showed weak growth on the SD/-Ade/-Leu/-Trp/-His medium supplemented with 30 mM 3-AT and 10 μM X-α-gal; these strains produced only a slight blue colour. Yeast cells expressing ZmICK5 or ZmICK6 combined with any of the cell cycle proteins could not grow on the auxotrophic SD media or hydrolyse X-α-gal. These results indicate that ZmICK1, ZmICK2, ZmICK3, and ZmICK4 are able to interact with ZmCDKA1 directly, whereas ZmICK7 can interact with ZmCycD2. Neither ZmICK5 nor ZmICK6 can interact with ZmCDKA1, ZmCycD1, ZmCycD2, or ZmCDKB.

Based on the alignment of maize A-type CDK protein sequences shown in Figure S4, further yeast two-hybrid analysis of the ZmICKs and A-type ZmCDKA was conducted. The results shown in Figure S6 indicate that the yeast expressing group C ZmICKs combined with ZmCDKA3 could grow normally and activate the expression of MET1 to hydrolyse the X-α-gal when grown on SD/-Ade/-Leu/-Trp/-His medium. Yeast cells expressing ZmICK7 in combination with ZmCDKA3 showed the same ability to grow normally on SD/-Ade/-Leu/-Trp/-His medium supplemented with 30 mM 3-AT, and this strain could degrade X-α-gal. However, both of yeast cells expressing ZmICK5 and ZmICK6 could not grow normally on SD/-Ade/-Leu/-Trp/-His medium and hydrolyse X-α-gal.

**Figure 5:**
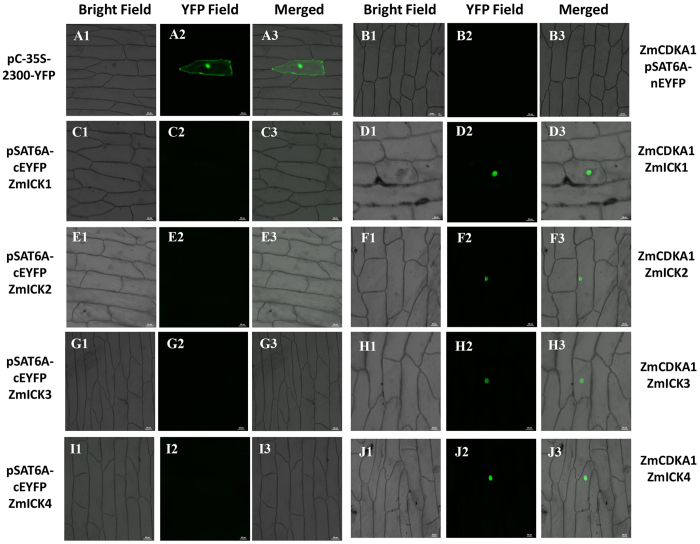
Bimolecular fluorescence complementation analysis of ZmICK1, ZmICK2, ZmICK3, and ZmICK4 with ZmCDKA1, the pCAMBIA2300-eYFP served as a positive control, the pSAT6A-cEYFP and pSAT6-ZmICK-nEYFP, and pSAT6A-ZmCDKA-cEYFP and pSAT6-nEYFP was used as a negative control. The pSAT6-ZmICK-nEYFP and the corresponding pSAT6-ZmICK-nEYFP and pSAT6A-ZmCDKA1-cEYFP images are shown side-by-side. (A1-J1) are the detection result under bright-field. (A2-J2) present the detection under the YFP field. (A3-J3) are the merged images of bright field and YFP field. Each alphabet labelled 1 to 3 represents the same combination. Bar = 50 μm.

Bimolecular fluorescence complementation was used to further analyse the interactions between Group C ZmICKs and the ZmCDKA1 and ZmCDKA3 proteins, the results of which are shown in Fig. 5 and Figure S7. In this analysis, the presence of EYFP in the nucleus of onion epidermal cells indicated that all of the group C ZmICKs could interact with ZmCDKA1 in the nucleus. Although interaction between the group C ZmICKs and ZmCDKA3 could be detected, the fluorescent signal in the nucleus of the onion epidermal cells was not strong. Nonetheless, the detection of fluorescence only in the nucleus is a positive indicator of the location of the interaction between these two proteins and is consistent with the sub-cellular localization and co-localization results.

### Group C ZmICKs affect CDKA1 function in fission yeast

**Figure 6:**
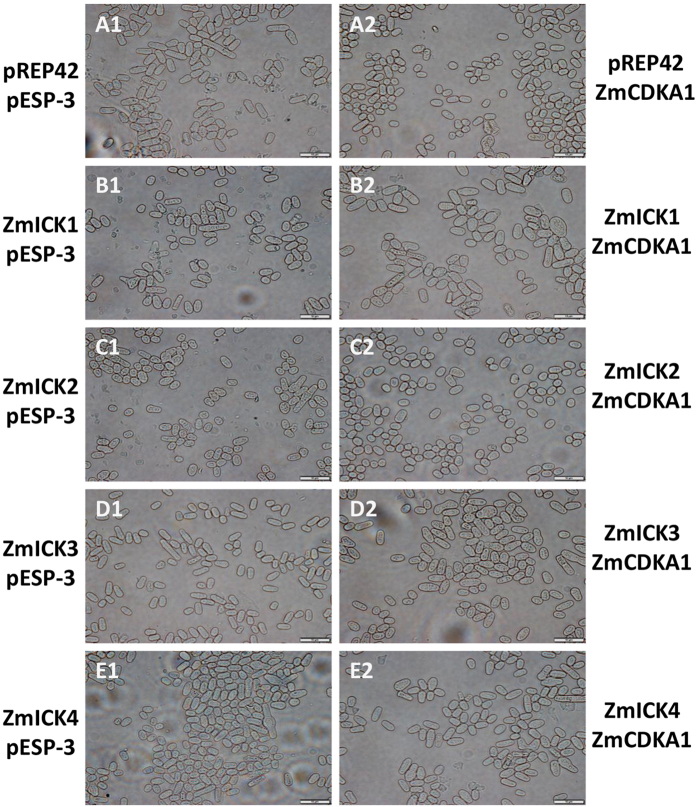
Cytological analysis of fission yeast cells expressing C-group ZmICKs, ZmCDKA and a combination of both. A1 represents wild-type fission yeast cells transformed with pREP42 and pESP-3. A2 shows yeast cells expressing ZmCDKA1 and pREP42. B1 to E1 presents the yeast cells expressing the C-group ZmICKs with pESP-3. B2 to E2 shows the yeast cells co-expressing C-group ZmICKs and ZmCDKA1. The ZmICK-pREP42 and the corresponding ZmICK-pREP42 and ZmCDKA1-pESP-3 images are shown side-by-side. Bars = 10 μm.

The biological roles of Group C ZmICKs were assayed in fission yeast *in vivo*. The group C *ZmICKs, ZmCDKA1*, and *ZmCDKA3* were each sub-cloned into an expression vector under the control of the thiamine-repressible promoter nmt1, after which they were introduced into yeast cells in different combinations. Previous research on *Arabidopsis* CDKA;1 in fission yeast cells indicated that the expression of dominant mutant CDKA;1 caused accelerated cell division, with the resulting cells appearing smaller than the control cells as a result of increased CDK activity^37^,^38^. As shown in Fig. 6 and statistical result (Figure S9), cells expressing *ZmCDKA1* and *ZmCDKA3* produced significantly smaller and rounder cells following division compared with cells transformed with control vector. Meanwhile, the cellular expression of Group C ZmICKs alone result in reduce in cell size compared with the control cells. When *ZmICKs* were co-expressed with *ZmCDKA1*, the yeast cell size was restored to the normal size in case of ZmICK3 and ZmICK4 and became even larger than normal yeast cells in case of ICK1. However, the cells remained small when ICK2 and ZmCDKA1 were both expressed. These results indicate that ZmICK1, ZmICK3 and ZmICK4 can inhibit the function of ZmCDKA1 in the fission yeast and ZmICK2 can not affect the function of ZmCDKA1 in fission yeast. Meanwhile, transformed and expressed *ZmICK1* with *ZmCDKA1* can promote cells enlargement significantly.

Over-expressed ZmCDKA3 also have an effect on fission yeast cell as shown on the Figure S8 and statistics data (Figure S9). When *ZmICKs* were co-expressed with *ZmCDKA3*, the yeast cell size was restored to the normal size in case of ZmICK2 and ZmICK4. However, the cells remained small in case of ZmICK1 and ZmICK3. Those results indicate that ZmCDKA3 also have influences on fission yeast cell and ZmICK2 and ZmICK4 show effect on the function of ZmCDKA3.

## Discussion

Maize ICK/KRP family protein sequences were identified through comparative analyses based on the *Arabidopsis* and rice ICK/KRP sequences using the Hmmer model and BLASTP. Using the conserved C-terminal region shared by *Arabidopsis* and rice ICK/KRPs^3^,^4^,^6^,^7^,^8^,^9^, nine genes containing a conserved C-terminal region corresponding to a CDI were identified. Two of these genes shared the same gene structure, the same CDS, and the same protein sequence; these genes were named *ZmICK6.1* and *ZmICK6.2*. As shown in Figure S10, alignment of 1000 bp of the upstream sequence indicated that these genes may have the same regulatory mechanism. When the *ZmICK* genes were cloned, *ZmICK8* did not encode a complete protein containing the CDI region despite a complete protein predicted by the reference genome. As the conserved C-terminal region is required for CDK-inhibiting function^16^, *ZmICK8* is considered a pseudo member of the maize ICK/KRP family, similar to OsICK7. OsICK7 lacks the conserved C-terminal CDK- and cyclin-binding region but shares similarities with other rice ICK/KRP sequences in the N-terminal region^8^ and is also considered a pseudogene.

Previous investigations of the ICK/KRP genes in various plant species have divided the plant ICK/KRPs into three classes^33^. In this study, the maize ICK/KRP genes were classified into class B and class C. Group C contains *ZmICK1* to *ZmICK4. ZmICK5, ZmICK6*, and *ZmICK7* belong to group B, which only contains monocotyledon ICK/KRP genes^33^. The phylogenetic analyses indicated that *ZmICK5, ZmICK6*, and *ZmICK7* were closely related to *OsICK1* and *OsICK2*. In a previous study, *OsICK1* and *OsICK3* were shown to be important for rice endosperm development and could affect seed filling^7^,^21^, suggesting that *ZmICK6* and *ZmICK7* might be involved in the development of the maize endosperm and seed filling. *ZmICK1* and *ZmICK5* were identified in maize endosperm in 2005^10^; the same study also indicated that both of these genes are associated with the development of the endosperm. In this study, *ZmICK1* and *ZmICK5* were classified into different groups, with *ZmICK1* belonging to class C and *ZmICK5* belonging to class B. However, previous research on ICK genes has also indicated the possibility of a complementary function^10^.

The function of a gene depends on various factors, including gene expression, the location of the protein, and the proteins that directly or indirectly interact with the target protein. ICK/KRP gene expression showed specificity in the timing and location of expression for different functions in different tissues of other plants^4^,^5^,^7^,^20^,^21^,^31^,^32^. The expression pattern of the seven maize ICK/KRP genes showed that they were expressed in a time- and tissue-specific manner. Most of the maize ICK/KRP genes were expressed at different levels in all of the tissues examined. *ZmICK1* and *ZmICK5*, which were previously identified in maize endosperm^10^, were highly expressed in the maize seed. However, all the ZmICKs were also expressed in the roots, stems, and leaves. Expression analysis of the ICK/KRP genes in *Arabidopsis* and poplar indicated that these genes are expressed in most tissues, and that there is no single specific expression pattern^33^. In rice, *Orysa;ICK1* is also the predominant rice ICK/KRP expressed during seed germination^7^. *Orysa;ICK5* and *Orysa;ICK6* were expressed during the early stages of panicle development^33^. *Orysa;ICK3* is strongly expressed in the caryopsis 2 days after flowering^21^. Moreover, the expression of ICK/KRP genes is also induced or suppressed by different plant hormones and salt stress^13^,^20^,^39^,^40^. The expression pattern of the ZmICKs indicated that ZmICKs may be associated with tissue development. When multiple genes from the same family are expressed in the same tissue, the difficulty in studying the function of these genes increases due to functional redundancies.

The cellular localization of the maize ICK/KRP proteins revealed that the seven ZmICKs mainly localize to the nucleus. Similar results have been found in *Arabidopsis*, rice, and tobacco ICK/KRPs^7^,^12^,^14^,^19^,^21^. A deletion study involving *Arabidopsis* ICK/KRPs indicated that there are several NLSs in those proteins, including the NLS YLQLRSRRL^14^,^18^, which has also been found in four OsiICK proteins^9^. Prediction analysis of the maize ICK/KRP proteins indicated that the four ZmICKs in group B contained a putative nuclear localization sequence (NLS) but did not contain the YLQLRSRRL sequence. However, conserved motif analysis of the ZmICKs in group C showed that motif 2 contained the YLELRSRRL sequence, which is similar to YLQLRSRRL. There is only one amino acid difference between YLELRSRRL and YLQLRSRRL: glutamine (Q) to glutamic acid (E). The YLELRSRRL sequence may permit the localization of the ICK/KRP proteins to the nucleus. In group B, motif 2 also contained the conserved sequence YIHLRSRML, which differs from the YLQLRSRRL sequence by only three amino acids. This sequence may also direct the ICK/KRPs to the nucleus. The ICK/KRP cellular localization results indicated that the ZmICKs were present in the nucleus; however, we could not confirm a functional relationship between the putative NLSs and ICK/KRP nuclear localization.

The ICK/KRP proteins bind to CDK complexes, which consist of CDK, cyclins, and other regulatory proteins. Thus, it is important to determine whether the different ICK/KRPs could interact with various CDKs and cyclins. Previous studies in maize have indicated that ZmICK1 and ZmICK5 can inhibit the activities of CDK and can bind with cyclin A1;3 and cyclin D5;1, which are associated with CDK activity, but not cyclin B1;3^10^,^23^. Studies in *Arabidopsis* and rice have shown that ICK/KRPs can bind directly to CDKA and the D-type cyclins. Deletion studies using a yeast two-hybrid system have indicated that the conserved C-terminus is critical for ICK interaction with other proteins^5^,^20^,^21^. However, the N-terminal region can interact with CYCD3;1 and has been shown to be a negative regulator of ICK1 in *Arabidopsis*^15^. Our study of maize ICKs also found that all seven ICK proteins contain the conserved motifs 1 and 3, which comprise the conserved C-terminus and are important for interactions with other cell cycle regulators. Based on the yeast two-hybrid and bimolecular fluorescence complementation assays, ZmICK1, ZmICK2, ZmICK3, and ZmICK4 can interact directly with ZmCDKA1 and ZmCDKA3, and ZmICK7 can interact with ZmCycD2. The ZmICKs could not interact with ZmCDKB, which is consistent with studies conducted in rice and *Arabidopsis*^6^,^21^.

Protein interactions may have an effect on the function of the protein. Yeast cells expressing *ZmCDKA1* produced significantly smaller and rounder cells following division compared with cells transformed with control vector suggest that maize A type CDK increases the CDKA activity in these fission yeast cells. The same results were obtained in previous research on Arabidopsis CDKA;1 in fission yeast cells^37^,^38^. However, the cellular expression of Group C ZmICKs alone result in reduce in cell size compared with the control cells transformed with vector, and it is in contrast to OsKRP3 in fission yeast^21^. Transformed and expressed the *ZmICK* genes and *ZmCDKA1* in fission yeast together restoring fission yeast cell phenotype indicated that ZmICK1, ZmICK3, and ZmICK4 can directly affect the function of kinase. ZmICK1 can interact directly with ZmCDKA1 and can affect the function of ZmCDKA1 in fission yeast shared a same result with previous research^10^,^23^. Combination of ZmICK1 and ZmCDKA1 can significantly enlarge fission yeast cell and the specific molecular mechanisms are unclear. However, overexpression of KRP2 in Arabidopsis also found the mature transgenic leaves consisted of enlarged cells and cell size may be increased by inhibition of cell cycle progression^4^. Although ZmICK2 could bind to ZmCDKA1 directly, this interaction had no obvious effect on the function of ZmCDKA1. ZmCDKA3 also has an effect on the division of fission yeast based on the statistical result and ZmICK2 and ZmICK4 could restore cell morphology caused by the ZmCDKA3.

In conclusion, this study identifies the ICK/KRP gene family in maize and provides basic information regarding maize ICK/KRPs and a characterization of their functions. The ZmICKs can be divided into two groups with specific characteristics based on their gene structures, a phylogenetic analysis, and their conserved motifs. The functions of some motifs have been identified. Motif 2 contains a sequence, YLELRSRRL, which is similar to the NLS sequence YLQLRSRRL^14^,^18^. Motifs 1 and 3 comprise the CDI region, which is important for the ICK/KRP interactions with A-type CDKs and cyclins^5^,^16^,^20^,^21^. The subcellular localization and co-localization analysis indicate that the ZmICKs locate in the nucleus and share the same location with A-type CDKs. Interaction analysis and the result on yeast cell size from the co-expression of the ZmICK genes and ZmCDKA together in fission yeast indicate that ZmICK1, ZmICK3 and ZmICK4 can directly bind with ZmCDKA1 and may inhibit its function. According to the current study, the specific functions of the ZmICKs in maize are still unknown. The subcellular localization, expression, and interaction with cyclins and A-type CDKs suggest that the ZmICKs may play different roles in growth and development. However, understanding the differences among the various members of ICK/KRP gene family in maize developmental processes is still a challenge.

## Materials and Methods

### Plant materials and growth conditions

The maize inbred line 18-599 was bred by the Maize Research Institute of Sichuan Agricultural University and shows good resistance to diseases and stress, high multiplication rates and seed production, strong recombination ability and wide adaptability. Maize inbred line 18-599 was grown at the university farm during the summer of 2013 according to the local standards for maize production. When silks emerged, strict self-pollination was performed every afternoon. The roots, stems, and leaves were collected when the maize was in the initial jointing stage of growth. The pollen and filaments were collected after the maize was in the tasselling period but before the filaments had emerged from the husks. The pericarp, embryo, and endosperm were isolated from the seeds 15 days after pollination. All samples were collected in the afternoon and were immediately frozen in liquid nitrogen and stored at −70 °C until use.

### Identification of putative ICK/KRP genes in the maize genome

To identify all putative ICK/KRP proteins in the maize genome, we utilized a hidden Markov model profile (http://hmmer.janelia.org/)^35^ and performed BLAST searches using the maize proteome sequence (ZmB73_5b_FGS_translations.fasta.gz) from the Maize Genome Sequence Project (http://ftp.maizesequence.org/current/filtered-set/)^34^ database and the known ICK/KRP protein sequences from rice and *Arabidopsi*s as the query. The ICK gene family information from *Arabidopsis* and *Oryza sativa* is listed in Table S2. We set our expectation cutoff based on the hmmscan of the *Oryza sativa* protein sequences, and the E-value chosen in HMMER was 1e^−10^ for cyclin-dependent kinase inhibitors (CDI) in maize. The length, pI, and MW of the maize ICK/KRP candidate proteins were computed using ExPASy (http://web.expasy.org/compute_pi/).

### Comparative analysis of the conserved motifs and gene structure

Conserved motifs were investigated based on multiple sequence alignment analyses using ClustalW and MEME (http://meme.nbcr.net/meme/cgi-bin/meme.cgi). Based on previous studies^33^, the minimum motif width, maximum motif width, and number of different motifs were specified as 6, 50, and 10, respectively, and the results were curated manually. To define the intron-exon arrangement of the ICK/KRP genes of the six species, both the gene sequence and the corresponding sequence from the reference genome were loaded into the Gene Structure Display Server (http://gsds.cbi.pku.edu.cn/). For better comparison and visualization, we removed the 5′ UTR and simplified the results.

### Sequence analysis and construction of the evolutionary tree

A multiple sequence alignment analysis was performed using ClustalW. Evolutionary trees were constructed using MEGA 5.10 software. The neighbour-joining (NJ) method was used with a Poisson correction model and 1000 bootstrap replicates.

### Cloning and sequence analysis of the maize cell cycle genes

Total RNA was isolated from root, stem, leaf, filament, anther, and seed 10 days after pollination and the embryo, endosperm, and pericarp of the seed 15 days after pollination of the inbred line 18-599 using TRIzol reagent (Invitrogen) according to the manufacturer’s instructions. First-strand cDNA synthesis was performed using 2 μg of total RNA and the PrimeScript^TM^ RT reagent kit with gDNA Eraser (TaKaRa, Dalian, China). All related genes were first cloned using KOD enzymes (Toyobo, Osaka, Japan). The maize cell cycle genes, D-type cyclins (AF351190.1; AF351189.1^41^), A-type CDKs (M60526.1, ZmCDKA1; GRMZM2G143213, *ZmCDKA2*^42^; GRMZM2G174596, ZmCDKA3^36^), and B-type CDK (GRMZM2G495626)^36^) were cloned from the cDNA using gene-specific primers containing NdeI and BamHI restriction sites. The putative ICK/KRP genes were cloned with primers containing NdeI and SalI restriction sites (Table S3). The amplified cDNA products were cloned into the pMD-19T vector (TaKaRa, Dalian, China), and the resulting plasmids were verified by sequencing.

### Expression analysis by semi-quantitative RT-PCR

RNA freshly extracted from different tissues (1.5 μg) was used as a template for the synthesis of first-strand cDNA. Primers used for semi-quantitative RT-PCR, which are listed in Table S3, were named qICKxF and qICKxR. Premix Taq (TaKaRa, Dalian, China) was used for the PCR reaction. To ensure the linearity of the results, the RT-PCR reactions were performed for no more than 32 cycles. PCR was performed under the following conditions: cDNA was pre-denatured at 94 °C for 4 min, followed by 30 to 32 cycles of 30 s at 94 °C, 30 s at 59 °C and 30 s at 72 °C in a 20 μL reaction mixture containing 10 μl of 2 × Premix Taq, 8 μl of H_2_O, 1 μl of template, 0.5 μl of sense primer, and 0.5 μl of anti-sense primer. All PCR reactions were repeated using at least three independent samples. The PCR products were fractionated on a 1.5% agarose gel containing GoldView™ and were photographed under UV light.

### Prediction of ICK/KRP cellular localization using PSORT II and confirmation with enhanced green fluorescent protein localization

Putative ZmICK nuclear localization signals (NLSs) were predicted using PSORT II (http://psort.ims.u-tokyo.ac.jp/), which was designed to identify nuclear localization signals within sequences^43^.

The sub-cellular localization of ZmICKs was further analysed through the transient expression of a fusion construct containing enhanced green fluorescent protein (eGFP) in *Nicotiana benthamiana*. To prepare the ZmICK-eGFP fusion constructs, the complete *ZmICK1* to *ZmICK7* coding sequences without the termination codons were digested with KpnI and XbaI (*ZmICK3* was digested with XbaI and SalI) (TaKaRa, Dalian, China) and then sub-cloned into the pCAMBIA2300-35S-eGFP expression vector^44^. The primers are listed in Table S3. Then, the plasmids were transformed into *Agrobacterium tumefaciens* strain EHA105, which was used to infect *Nicotiana benthamiana*. The subcellular localization of the GFP fusion proteins was visualized with an ECLIPSE 80i florescence microscope under excitation with blue light at 488 nm.

### Cellular co-localization analysis of C-type ZmICKs and A-type CDKs

The cellular co-localization analysis of the ZmICKs and A-type CDKs was conducted by transiently expressing fusion constructs containing enhanced green fluorescent protein (eGFP) and red fluorescence protein (RFP) in onion epidermal cells. To prepare the ZmICK-eGFP fusion constructs, the complete coding sequences of *ZmICK1, ZmICK2*, and *ZmICK4* lacking the termination codons were digested with KpnI and XbaI, while *ZmICK3* was digested with XbaI and SalI. The sequences were then sub-cloned into the expression vector pCAMBIA2300-35S-eGFP. To prepare the A-type CDK-RFP fusion constructs, the complete coding sequence of A-type CDK lacking the termination codon was digested with KpnI and BamHI and then was sub-cloned into the pCAMBIA2300-35S-RFP expression vector. The primers are listed in Table S3. The cellular co-localization of the eGFP fusion proteins and the RFP fusion protein was visualized using an ECLIPSE 80i florescence microscope under different excitation wavelengths.

### Yeast two-hybrid analysis of ICK/KRP proteins and cell cycle-related proteins

The interactions between the ICK/KRP proteins and different maize cyclins and CDKs were analysed using the GAL4 yeast two-hybrid system (Clontech). The CDS of the ZmICK genes were cloned into pMD19-T and digested with NdeI and SalI, whereas *ZmCDKA1, ZmCDKB, ZmCycD1*, and *ZmCycD2* were digested with NdeI and BamHI. The primers used are listed in Table S3. The purified gene fragments were cloned into vectors containing the yeast two-hybrid DNA-binding domain (pGBKT7, BD) and the activation domain (pGADT7, AD). All the resulting plasmids were confirmed by sequencing. Different combinations of the BD and AD constructs were introduced into AH109 using a lithium acetate transformation method. The positive and negative controls used pGADT7-T co-transformed with pGBKT7-53 or pGBKT7-lam, respectively. The transformants were first incubated on synthetic dextrose (SD) media lacking tryptophan (Trp) and leucine (Leu) for 3 days. Then, a single clone was grown in 1 ml of SD/-Trp/-Leu for 12 hours, and 6 μl of the culture was grown on synthetic dextrose (SD) medium lacking tryptophan (Trp), leucine (Leu), adenine (Ade), and histidine (His) supplemented with 30 mM 3-AT for 3 days. Finally, 10 μM 5-bromo-4-chloro-3-indolyl-α-D-galactopyranoside (X-α-gal) was smeared onto the plate to detect α-galactosidase activity.

### Bimolecular fluorescence complementation analysis of the interactions between C-type ZmICKs and ZmCDKA1 and ZmCDKA3

Bimolecular fluorescence complementation analysis of C-type ZmICKs with A-type CDKs was conducted by transiently expressing fusion constructs containing N-terminal enhanced yellow fluorescent protein (EYFP, pSAT6-nEYFP) and C-terminal enhanced yellow fluorescent protein (EYFP, pSAT6A-cEYFP) in onion epidermal cells^45^,^46^. To prepare the ZmICK-nEYFP fusion constructs, the complete coding sequence of C-type *ZmICKs* was digested with EcoRI and SmalI and sub-cloned into pSAT6-nEYFP. A-type CDKs were digested with EcoRI and SmalI and then sub-cloned into the pSAT6A-cEYFP expression vector to generate the ZmCDKA-cEYFP fusion construct. The primers used are listed in Table S3. pSAT6A-cEYFP plus pSAT6-ZmICK-nEYFP and pSAT6A-ZmCDKA-cEYFP plus pSAT6-nEYFP were used as negative controls. The bimolecular fluorescence complementation of the EYFP fusion proteins was visualized using an ECLIPSE 80i florescence microscope under excitation with yellow light at 488 nm.

### Expression of the C-type ZmICK genes, *ZmCDKA1*, and *ZmCDKA3* in fission yeast

The *ZmICK* fragments were cloned into the fission yeast expression vector pREP42 (Addgene) under the control of the thiamine-repressible promoter nmt41. *ZmCDKA1* and *ZmCDKA3* were sub-cloned into the fission yeast expression vector pESP-3 (Stratagene) downstream of the thiamine-repressible promoter nmt1. The primers used in this experiment were the same as the primers used in the yeast two-hybrid assay and are listed in Table S3. The PEG-LiAc method was used to transform *S. pombe* strain FY12137 (h90 ura4-D18 leul-32) (National BioResource Project Yeast Genetic Resource Center [NBRP/YGRC]) with different combinations of the plasmids: pREP42/pESP-3, ZmICKs-pREP42/pESP-3, pREP42/ZmCDKA-pESP-3, and ZmICKs-pREP42/ZmCDKA-pESP-3^21^. The transformants were first incubated on synthetic dextrose (SD) media lacking uracil (Ura) and leucine (Leu) for 4 days, after which a single clone was picked and inoculated into 1 ml of liquid SD/-Ura/-Leu for 60 hours. The morphology of the yeast cells in the culture was examined with a phase contrast microscope. The relative cell area of fission yeast was measured through surrounding yeast cell area with a perimeter in the area selection tools of the image J. All of those cells in the five views were measured about each treatment of the fission yeast. The test of actual cell area (um^2^) were calculated based on the scaleplate of the picture and significance testing were determined by the paired *t*-test (P = 0.05). The averages and variability were determined by Excel 2007.

### Data Availability

The data reported in this article have been deposited in National Center for Biotechnology Information (http://www.ncbi.nlm.nih.gov). The accession number of ZmICK7 and ZmICK8 are KX643357 and KX643358.

## Additional Information

**How to cite this article:** Xiao, Q. *et al*. Identification and functional analysis of the ICK gene family in maize. *Sci. Rep.* **7**, 43818; doi: 10.1038/srep43818 (2017).

**Publisher's note:** Springer Nature remains neutral with regard to jurisdictional claims in published maps and institutional affiliations.

## Supplementary Material

Supplementary Information

Supplementary Dataset
