# Supplementary material for: Identification and functional analysis of the ICK gene family in maize
Source: Sci Rep. 2017 Mar 6;7:43818. doi: 10.1038/srep43818 (PMC5338338; doi:10.1038/srep43818)
Supplement: Supplementary Information [file srep43818-s1.pdf]

**Identification and functional analysis of the ICK gene family in maize**

Qianlin Xiao[1], Chunxia Zhang[1], Hui Li[1], Bin Wei[1], Yongbin Wang[1], Huanhuan Huang[1], Yangping Li[1], Guowu Yu[1], Hanmei Liu[2], Junjie Zhang[2], Yinghong Liu[3], Yufeng Hu[1]*, and Yubi Huang[1]*

[1] College of Agronomy, Sichuan Agricultural University, Chengdu 611130, Sichuan, China

[2] College of Life Science, Sichuan Agricultural University, Ya'an 625014, Sichuan, China

[3] Maize Research Institute, Sichuan Agricultural University, Chengdu 611130, Sichuan, China

***To whom correspondence should be addressed.**

Email: yubihuang@sohu.com; huyufeng@sohu.com

Tel: 86-028-86290868; Fax: 86-028-86290870

E-mail addresses of the authors: Qianlin Xiao: 420479227@qq.com; Chunxia Zhang: 458167898@qq.com; HuiLi: 517285740@qq.com; Bin Wei: gwbyxu@gmail.com; Yongbin Wang: wwyybb007@qq.com; Huanhuan Huang: 14935483@qq.com; Yangping Li: yangpingli103@gmail.com; Guowu Yu: 2002ygw@163.com; Hanmei Liu: hanmeil@163.com; Junjie Zhang: junjiezh@163.com; Yinghong Liu: 18926348@qq.com; Yufeng Hu: huyufeng@sohu.com; Yubi Huang: yubihuang@sohu.com

## List of Supplementary Information

**Table S1.** The basic information about the identified ICK/KRP candidate genes in Maize. Length of bp* means the length of cDNA; Length of aa* shows the number of amino acid; In/ex means the number of Intron-exon; Mw is the molecular weight of the protein; Chr represents the chromosome.

**Table S2.** The basic information about the ICK/KRP gene family of *Arabidopsis* and *Orysa sativa*. Length of bp* means the length of cDNA; Length of aa* shows the number of amino acid; In/ex means the number of Intron-exon; Mw is the molecular weight of the protein; Chr represents the chromosome; Location shows the detail location on the chromosome.

**Table S3.** Primers for the main research. PCR annealing temperature is 57 ℃ to 61 ℃ for all genes based on the primers information. "Category" indicates the carriers constructed and expression analysis in research, "Gene Name" represents the name of gene in the article, "Primer name" and "Primer Sequence" represents the primer name and sequence of related gene in this article.

**Table S4.** The conserved motifs sequence information of ICK in maize.

**Table S5.** The Nuclear Location Signal (NLS) predicted information about the ICK/KRP putative proteins in Maize. The table contained putative protein sequence accession number, protein name, putative NLS, putative NLS model and certainty.

**Fig. S1** Physical locations information about maize ICK genes, the grey boxes on chromosomes indicate the approximate locations of the 9 maize ICK genes. CHR is the abbreviation of chromosomes and represents as black bars. The number corresponds to the number of chromosomes.

**Fig. S2** The primary sequence analysis of 40 ICK/KRP genes from *Arabidopsis thaliana* (*AtICK*), *Oryza sativa* (*OsICK*), *Brachypodium distachyon* (*BdKRP*), *Glycine max* (*GmKRP*), *Populus trichocarpa* (*PtKRP*) and *Zea mays* (*ZmICK*) contains phylogenetic analysis, gene

structure and motif analysis. The different color boxes represent the different motif in the motif analysis. The three main components in gene structure analysis represent CDS region, upstream/downstream and Intron, respectively.

**Fig. S3** The results from the analysis of the cloned ZmICK genes. (A) The roots, stems and leaves were collected when maize bred line 18-599 was in the initial jointing stage of growth. (B) and (C) are differences between the cloned sequences from maize bred line 18-599 and the reference sequence of B73. ZmICK7 has a three base pair insertion and led to a single amino acid inserted. ZmICK8 contains a 109bp inserted fragment led to an early termination of translation and lost the conserved CDI region. (D) Conserved motif analysis of the cloned ZmICK proteins sequence. Different coloured boxes represent the conserved motifs, the length of the long black lines represents the amino acid number of the ICK protein sequences, the short black line above the boxes or the long black line represents the predicted nuclear localization signals, and the blue boxes indicate the conserved domain CDI regions.

**Fig. S4** The protein sequence alignment between three A-type CDKs of maize and Cdc2 of S.pombe. The specific amino acid sites are marked on the sequence alignment including the conserved S-TKc domain, ATP binding site, Cyclin-binding site and T-loop region.

**Fig. S5** Co-localization of C-group ZmICKs and A-type CDKs. Combinations of the ZmICK-eGFP fusion protein vectors and the CDKA-RFP fusion protein vectors were transformed into onion epidermal cell with a gene gun and visualized with an ECLIPSE 80i fluorescence microscope. A is the co-localization about control pCAMBIA2300-35S-eGFP and pCAMBIA2300-35S-RFP. B is the co-localization result of fusion protein ZmICK1-eGFP with ZmCDKA-RFP. C shows the co-localization result of fusion protein ZmICK2-eGFP with ZmCDKA-RFP. D indicates the co-localization result of fusion protein ZmICK3-eGFP with ZmCDKA-RFP. E presents the co-localization result of fusion protein ZmICK4-eGFP with ZmCDKA-RFP. The images were detected under a bright-field, RFP, or GFP view and were then merged three into one. Bar=50 μm.

**Fig. S6** The results of the yeast two-hybrid analysis of ZmICKs and ZmCDKAs.

SD/-Leu/-Trp indicates media lacking tryptophan (Trp) and leucine (Leu).

SD/-Ade/-His/-Leu/-Trp represents the synthetic dextrose (SD) media lacking tryptophan (Trp), leucine (Leu), adenine (Ade) and histidine (His).

SD/-Ade/-His/-Leu/-Trp/30mM3-AT/10uM X-a-gal indicates the synthetic dextrose (SD) media contain 30 mM 3-AT and 10 uM X-a-gal. The gray box indicates the growth condition on the same medium and every yeast plaque indicates the growth of the transformants. "+" represents the positive control of the yeast two-hybrid, the combination of pGADT7 and pGBKT7-53. "-" shows the negative control of yeast two-hybrid, the pGADT7-T co-transformed with pGBKT7-lam.

**Fig. S7** Bimolecular fluorescence complementation analysis of ZmICK1, ZmICK2, ZmICK3, and ZmICK4 with ZmCDKA3, the pCAMBIA2300-eYFP served as a positive control, the pSAT6A-cEYFP and pSAT6-ZmICK-nEYFP, and pSAT6A-ZmCDKA3-cEYFP and pSAT6-nEYFP was used as a negative control. The pSAT6-ZmICK-nEYFP and the corresponding pSAT6-ZmICK-nEYFP and pSAT6A-ZmCDKA3-cEYFP images are shown side-by-side. (A1 - J1) are the detection result under bright-field. (A2-J2) present the detection under the YFP field. (A3-J3) are the merged images of bright field and YFP field. Each alphabet labelled with 1 to 3 represents the same combination. Bar=50 μm.

**Fig. S8** Cytological analysis of fission yeast cells expressing C-group ZmICKs, *ZmCDKA3* and a combination of the proteins. A1 represents wild-type fission yeast cells transformed with pREP42 and pESP-3. A2 shows yeast cells expressing *ZmCDKA3* and pREP42. B1 to E1 presents the yeast cells expressing the C-group ZmICKs-pREP42 with pESP-3. B2 to E2 shows the yeast cells co-expressing C-group ZmICKs and *ZmCDKA3*. The ZmICKs-pREP42 and the corresponding ZmICKs-pREP42 with ZmCDKA3-pESP-3 images are shown side-by-side. Bars=10 μm.

**Fig. S9** The averages with standard deviations of the actual cell size ($um^2$). Averages are the average of the transformation of the same combination between different views and standard

deviations represent the degree of dispersion of them. The X-axis represents the actual cell area size ($um^2$). The Y-axis shows the different transformation of combination. "**" indicates significant differences in mean values between the different treatments and controls.

**Fig. S10** The 1000bp upstream sequence alignment between ZmICK6.1 and ZmICK6.2.

## Supplementary Table

**Table S1. The basic information about the identified ICK/KRP candidate genes in Maize.**

| Maize Sequence Gene ID | Gene Name | length bp* | length aa* | In/ex | pI | MW (kD) | Chr | Location | Reference | NCBI Accession No. | Synonyms |
|---|---|---|---|---|---|---|---|---|---|---|---|
| GRMZM2G116885 | *ZmICK1* | 1084 | 190 | 2/3 | 8.82 | 21.29 | 1 | 232,980,076-232,983,251 | Coelho et al, 2005 | NP_001105778 | *ZmKRP1* |
| GRMZM2G037926 | *ZmICK2* | 1275 | 192 | 2/3 | 8.94 | 21.399 | 5 | 29,641,580-29,644,988 | This study | NP_001168443 | |
| GRMZM2G157510 | *ZmICK3* | 1446 | 213 | 2/3 | 7.56 | 23.72 | 1 | 8,442,682-8,446,230 | This study | NP_001152562<br>NP_001183727 | |
| GRMZM2G358931 | *ZmICK4* | 1343 | 215 | 2/3 | 8.28 | 23.831 | 9 | 152,252,293-152,255,757 | This study | NP_001132400 | |
| GRMZM2G101613 | *ZmICK5* | 1277 | 253 | 3/4 | 8.41 | 25.689 | 5 | 209,770,434-209,772,179 | Coelho et al, 2005 | NP_001105779<br>NP_001149832 | *ZmKRP2* |
| GRMZM2G084570 | *ZmICK6.1* | 1267 | 263 | 3/4 | 8.65 | 26.673 | 4 | 178,774,992-178,776,727 | This study | NP_001147155 | |
| GRMZM5G854731 | *ZmICK6.2* | 1267 | 263 | 3/4 | 8.65 | 26.673 | 8 | 112,022,205-112,023,940 | This study | NP_001147155 | |
| GRMZM2G343769 | *ZmICK7* | 969 | 275 | 3/4 | 9.11 | 28.875 | 9 | 7,870,479-7,871,904 | This study | KX643357 | |
| GRMZM2G154414 | *ZmICK8* | 1316 | 216 | 1/2 | 9.09 | 23.09 | 4 | 5,410,267-5,411,691 | This study | KX643358 | |

**Table S2. The basic information about the ICK/KRP gene family of *Arabidopsis* and *Orysa sativa***

| Gene Locus | Gene Name | length bp* | length aa* | In/ex | Chr | Location | NCBI Accession No. | Synonyms |
|---|---|---|---|---|---|---|---|---|
| AT2G23430 | *Arath;ICK1* | 950 | 191 | 3/4 | 2 | 9976746-9978021 | NM_127907/NP_179924 | *Arath;KRP1* |
| AT3G50630 | *Arath;ICK2* | 979 | 209 | 3/4 | 3 | 18800380-18801587 | NM_114923/NP_190632 | *Arath;KRP2* |
| AT3G24810 | *Arath;ICK3* | 920 | 189 | 2/3 | 3 | 9060896-9061998 | NM_113393/NP_189125 | *Arath;KRP5* |
| AT3G19150 | *Arath;ICK4* | 908 | 196 | 3/4 | 3 | 6616352-6617696 | NM_112802/NP_188546 | *Arath;KRP6* |
| AT1G49620 | *Arath;ICK5* | 789 | 195 | 3/4 | 1 | 18365491-18366559 | | *Arath;KRP7* |
| AT5G48820 | *Arath;ICK6* | 669 | 222 | 2/3 | 5 | 19792270-19794408 | NM_124259/NP_199693 | *Arath;KRP3* |
| AT2G32710 | *Arath;ICK7* | 1411 | 289 | 2/3 | 2 | 13873436-13875831 | NM_128830/NP_565750 | *Arath;KRP4* |
| | | | | | | | | |
| OS02G0762400 | Orysa;ICK1 | 1265 | 262 | 3/4 | 2 | 32111311-32112948 | NM_001054739/NP_001048204 | *Orysa;KRP1* |
| OS06G0213700 | Orysa;ICK2 | 750 | 249 | 3/4 | 6 | 5794738-5795810 | | *Orysa;KRP2* |
| OS11G0614800 | Orysa;ICK3 | 1214 | 214 | 3/4 | 11 | 23866045-23867666 | NM_001074803/NP_001068271 | *Orysa;KRP3* |
| OS09G0459900 | Orysa;ICK4 | 917 | 142 | 2/3 | 9 | 17390977-17392630 | | *Orysa;KRP6* |
| OS03G0137800 | Orysa;ICK5 | 1045 | 221 | 2/3 | 3 | 2088673-2091654 | NM_001055440/NP_001048905 | *Orysa;KRP5* |
| OS10G0471700 | Orysa;ICK6 | 1025 | 194 | 2/3 | 10 | 17499846-17503202 | NM_001071364/NP_001064829 | *Orysa;KRP4* |

**Table S3. Primers for the research**

| Category | Gene Name | Prmier Name | Primer Sequence |
|---|---|---|---|
| Yeast two-hybrid system vector pGBKT7 | *ZmICK1* | ICK1-BDF/ICK1-BDR | 5'-3'CATATGATGGGCAAGTACATGCG/5'-3'GTCGACTCAGTCTAGCTTCACCCAC |
| | *ZmICK2* | ICK2-BDF/ICK2-BDR | 5'-3'CATATGATGGGCAAATACATGCG/5'-3'GGTCGACTCAGTCTAGCTTCACCC |
| | *ZmICK3* | ICK3-BDF/ICK3-BDR | 5'-3'CATATGATGGGGAAGTACATGCGC/5'-3'GTCGACCTAGCAGTCTAGCCTCGC |
| | *ZmICK4* | ICK4-BDF/ICK4-BDR | 5'-3'CATATGATGGGGAAGTACATGC/5'-3'GTCGACCTAACAGTCTAGCCTCGC |
| | *ZmICK5* | ICK5-BDF/ICK5-BDR | 5'-3'CATATGATGGGGAAGTACATGCGC/5'-3'GTCGACTCAGATGCTGACCACCG |
| | *ZmICK6* | ICK6-BDF/ICK6-BDR | 5'-3'CCATATGATGGGGAAGTACATGCG/5'-3'GTCGACTCAGATGCTGACCCCTG |
| | *ZmICK7* | ICK7-BDF/ICK7-BDR | 5'-3'CATATGATGGGGAAGTACATGAGG/5'-3'GTCGACTCACCAACCGCTGGC |
| Yeast two-hybrid system vector pGADT7 | *ZmCDKA1* (M60526.1) | CDKA1-ADF/CDKA1-ADR | 5'-3'GCATATGATGGAGCAGTACGAGAAGG/5'-3'TGGATCCTCACTGTACCACTTCAAGG |
| | *ZmCDKA3(GRMZM2G174596)* | CDKA3-ADF/CDKA3-ADR | 5'-3'CATATGATGGACCAGTACGAGAAGGTG/5'-3'ATCCATGGATCCCTAGGCGTGCT |
| | *ZmCDKB* (GRMZM2G495626) | CDKB-ADF/CDKB-ADR | 5'-3'TCGCATATGATGCCCTTGCCC/5'-3'AGTAGGATCCCTAGAACTGGGACTTGTC |
| | *ZmCycD1* (AF351190.1) | CycD1-ADF/CycD1-ADR | 5'-3'TCTGATTCATATGATGGGGGACG/5'-3'GGATCCCTACTGTCGCTGAGGTG |
| | *ZmCycD2* (AF351189.1) | CycD2-ADF/CycD2-ADR | 5'-3'CATATGATGGTGCCGGGCTATGAC/5'-3'GCGTAGGATCCTTAGAGTAGACGTCTAGTG |
| Cellular Location vector pC2300-35s-eGFP | *ZmICK1* | LICK1F/LICK1R | 5'-3'GGTACCATGGGCAAGTACATG/5'-3'TCTAGAGTCTAGCTTCACCC |
| | *ZmICK2* | LICK2F/LICK2R | 5'-3'GGTACCATGGGCAAATACATG/5'-3'TCTAGAGTCTAGCTTCACCC |
| | *ZmICK3* | LICK3F/LICK3R | 5'-3'TCTAGAATGGGGAAGTACATGCG/5'-3'GTCGACGCAGTCTAGCCTCG |
| | *ZmICK4* | LICK4F/LICK4R | 5'-3'GGTACCATGGGGAAGTACATGC/5'-3'TCTAGAACAGTCTAGCCTCGCCCAC |
| | *ZmICK5* | LICK5F/LICK5R | 5'-3'GGTACCATGGGGAAGTACATGC/5'-3'TCTAGAGATGCTGACCACC |
| | *ZmICK6* | LICK6F/LICK6R | 5'-3'GGTACCATGGGGAAGTACATGC/5'-3'TCTAGAGATGCTGACCCCT |
| | *ZmICK7* | LICK7F/LICK7R | 5'-3'GGTACCATGGGGAAGTACATGAGG/5'-3'TCTAGACCAACCGCTGGCTA |
| Semi-quantitative RT-PCR | *ZmICK1* | qICK1F/qICK1R | 5'-3'CGCAACTAAGGAGGCTGAG/5'-3'GTCATCTGGGTGTTAATCAAGC |
| | *ZmICK2* | qICK2F/qICK2R | 5'-3'ACGCCATCCCAAGTTCAAC/5'-3'CTATCAGTCTAGCTTCACCCATTC |
| | *ZmICK3* | qICK3F/qICK3R | 5'-3'ACTTCTGTCCCGTGAACGAC/5'-3'GCGGAGCTAGTCACATTGTG |
| | *ZmICK4* | qICK4F/qICK4R | 5'-3'GCAGGATGGAAACCTCAGTC/5'-3'CTCGCCCACTCATATCGAC |
| | *ZmICK5* | qICK5F/qICK5R | 5'-3'GCACGAGGTCCAGGTCC/5'-3'CAGGTGGATGTAGCAGCTTC |

| | | | |
|---|---|---|---|
| | *ZmICK6* | qICK6F/qICK6R | 5'-3'AAGACTGGCTGCTCGTCGTC/5'-3'CGAAGTCGAAGTTGTACTTGGAAG |
| | *ZmICK7* | qICK7F/qICK7R | 5'-3'GGCAGTGACTGGGAACACCAGC/5'-3'AGTCGTCGTCGTCGTTGCGTG |
| | *ZmCDKA1* (M60526.1) | qCDKA1F/qCDKA1R | 5'-3'CCTCGCCACTTAGTTCGTTCC/5'-3'TTGCCGTGGTTCATCTCCTT |
| | *ZmCDKA3(GRMZM2G174596)* | qCDKA3F/qCDKA3R | 5'-3'CTCCCCTGCTCCCTCTGTAA/5'-3'CTCTCTCCAGCCATCATACAGG |
| | *ZmCDKB* (GRMZM2G495626) | qCDKBF/qCDKBR | 5'-3'AGGCTCTCTTTCCTGGTGACTC/5'-3'TCCAGGCCACTGTTCCTCAG |
| | *ZmCycD1* (AF351190.1) | qCycD1F/qCycD1R | 5'-3'TTCACTTTTCTTCCCTTTCCC/5'-3'TTCATCCAGTCCTCCATTTCC |
| | *ZmCycD2* (AF351189.1) | qCycD2F/qCycD2R | 5'-3'TGTATTGATTGCTCGCTGCTC/5'-3'GGAACTCCGTCAAAATCCCAT |
| | *Actin* | ActinF/ActinR | 5'-3'TCACTACGACTGCCGAGCGAG/5'-3'GAGCCACCACTGAGGACAACATTAC |
| | *ZmCDKA1* (M60526.1) | CDKA1BiFCF/CDKA1BiFCR | 5'-3'GAATTCATGGAGCAGTACGAGAAG/5'-3'CCCGGGCTGTACCACTTCAAGGTC |
| | *ZmCDKA3(GRMZM2G174596)* | CDKA3BiFCF/CDKA3BiFCR | 5'-3'GAATTCATGGACCAGTACGAGAAG/5'-3'CCCGGGGGCGTGCTCGAGGTCCCT |
| Bimolecular Fluorescence Complementation | *ZmICK1* | ZmICK1BiFCF/ZmICK1BiFCR | 5'-3'GAATTCATGGGCAAGTACATGCGCA/5'-3'CCCGGGGTCTAGCTTCACCCACTCA |
| | *ZmICK2* | ZmICK2BiFCF/ZmICK2BiFCR | 5'-3'GAATTCATGGGCAAATACATGCGCA/5'-3'CCCGGGGTCTAGCTTCACCCATTC |
| | *ZmICK3* | ZmICK3BiFCF/ZmICK3BiFCR | 5'-3'GAATTCATGGGGAAGTACATGCGCA/5'-3'CCCGGGGCAGTCTAGCCTCGCCCA |
| | *ZmICK4* | ZmICK4BiFCF/ZmICK4BiFCR | 5'-3'GAATTCATGGGGAAGTACATGCGCA/5'-3'CCCGGGACAGTCTAGCCTCGCCCAC |
| | *ZmCDKA1* (M60526.1) | CDKA1-ADF/CDKA1-ADR | 5'-3'CATATGATGGAGCAGTACGAGAAGG/5'-3'TGGATCCTCACTGTACCACTTCAAGG |
| | *ZmCDKA3(GRMZM2G174596)* | CDKA3-ADF/CDKA3-ADR | 5'-3'CATATGATGGACCAGTACGAGAAGGTG/5'-3'ATCCATGGATCCCTAGGCGTGCT |
| Co-expressing the gene in the fission yeast cell | *ZmICK1* | ICK1-BDF/ICK1-BDR | 5'-3'CATATGATGGGCAAGTACATGCG/5'-3'GTCGACTCAGTCTAGCTTCACCCAC |
| | *ZmICK2* | ICK2-BDF/ICK2-BDR | 5'-3'CATATGATGGGCAAATACATGCG/5'-3'GGTCGACTCAGTCTAGCTTCACCC |
| | *ZmICK3* | ICK3-BDF/ICK3-BDR | 5'-3'CATATGATGGGGAAGTACATGCGC/5'-3'GTCGACCTAGCAGTCTAGCCTCGC |
| | *ZmICK4* | ICK4-BDF/ICK4-BDR | 5'-3'CATATGATGGGGAAGTACATGC/5'-3'GTCGACCTAACAGTCTAGCCTCGC |

**PCR annealing temperature is 57 ℃ to 61 ℃ for all genes based on the primers information**

**Table S4. The conserved motifs sequence information of ZmICKs**

| Protein Name | Motif 1 | Motif 2 | Motif 3 | Motif 4 | Motif 5 |
|---|---|---|---|---|---|
| ZmICK1 | HAVPSSREMNEYFAAEQRRQQQDFIDKYNFDPANDCPL | MGKYMRKAKASSEVVIMDVA | QTQWEEGAGGEYLELRNRRLEKLPPPPAT | MERITRETTPCSLIN | GRFEWVKL |
| ZmICK2 | HAIPSSTEMNEYFAAEQRRQQQAFIDKYNFDPVNDCPL | MGKYMRKVKVSSEVAIMDVA | QVQQEEGCGGEYLELRSRRLEKLPPQVAS | MGRNTRETTPCSLIN | GRFEWVKL |
| ZmICK3 | RFIPSSLEMEEFFSAAEQQEQHSFREKYNFCPVNDCPL | MGKYMRKGKVSGEVAVMEVP | DKGDAEDAAAEYLELRSRRLEKPHKEHPS | MERSTRETTPCSLIR | GRYEWARL |
| ZmICK4 | RFIPSSLEMEEFFSAAEQQEQHNFREKYNFCPVNDCPL | MGKYMRKGKMSGEVAVMEVP | DKGDADDAAGQYLELRSRRLEKPHKEHQP | MERSTRETTPCSLIR | |
| ZmICK7 | RATPPAEEIEQFFAAAEKAQAERFAAKYNFDVARGLPL | MGKYMRKRKRNVGRTTPAAE | NTSGAAAPGSCYLHLRTRRLQFLPGGEGE | CRRDRRETTPSSQAR | GRYEWTPV |
| ZmICK5 | LIVPPAHEIQEFFAAAEAAQAKRFASKYNFDFVRGVPL | MGKYMRKCRGAAGAEVAAVE | SVGAGGDGGSCYIHLRSRMLFMAPPQPQP | PDRERRETTPSSRAH | GRFEWAPV |
| ZmICK6.1 | LIVPPAQEIQEFFAAAEAAHAKRFASKYNFDFVRGVPL | MGKYMRKRRGAAGEGVAAVE | GAGGGDGGSCCYIHLRSRMLFMAAPQQQP | PDRERRETTPSSSRA | GRFEWTPG |
| ZmICK6.2 | LIVPPAQEIQEFFAAAEAAHAKRFASKYNFDFVRGVPL | MGKYMRKRRGAAGEGVAAVE | GAGGGDGGSCCYIHLRSRMLFMAAPQQQP | PDRERRETTPSSSRA | GRFEWTPG |
| ZmICK8 | PPPPTETEIEAFFADAELAERRRFAEAYNYDVALDRPL | | | | GRFEWVPL |

| Protein Name | Motif 6 | Motif 7 | Motif 8 | Motif 9 | Motif 10 |
|---|---|---|---|---|---|
| ZmICK1 | LGVRTRARALALQR | MTSTPGSTRSGHSCH | SYGENMLELE | | TRRSGGRK |
| ZmICK2 | LGVRTRARALALQR | MISTPGSTTRSSHSS | SYGENMLESE | | VRRCGGRK |
| ZmICK3 | LGVRTRSRTLALQR | MISTPGSTTKSKTSS | SFGDNVLDLD | | TKRGAGRK |
| ZmICK4 | LGVRTRSRTLALQR | MISTPGSTTKSKTSN | SFGENVLDLD | SRRRMETSVCR | AKRGAGRK |
| ZmICK7 | HGVRTRAQSDAVAS | PVEGAGSSRCSSTAS | | EARNDDDDYCG | TRHRRRRK |
| ZmICK5 | VGVRTRSRSAAATG | AALAAGLSRCSSTAS | AGGDHVLVDV | ELSDLESDLAG | KVVAPRRK |
| ZmICK6.1 | VGVRTRSRSAAATG | VALAAGLSRCSSTAS | VDGDHVPDVV | ELSDLESDLVG | KVAPPRRK |
| ZmICK6.2 | VGVRTRSRSAAATG | VALAAGLSRCSSTAS | VDGDHVPDVV | ELSDLESDLVG | KVAPPRRK |
| ZmICK8 | LGVGARQQQLCADD | | | KANKHENDECG | TRSRPRRH |

**Table S5. The information about the NLS prediction of ICK/KRP putative proteins in Maize**

| ACCESSION NO. | Protein Name | Putative NLS | Model | Certainty |
|---|---|---|---|---|
| GRMZM2G116885-T01 | ZmICK1 | no detect | n/a | Not Clear |
| GRMZM2G037926-T01 | ZmICK2 | no detect | n/a | Not Clear |
| GRMZM2G157510-T01 | ZmICK3 | no detect | n/a | Not Clear |
| GRMZM2G358931-T02 | ZmICK4 | no detect | n/a | Not Clear |
| GRMZM2G101613-T01 | ZmICK5 | 52 (5) RRKR | [K/R]4 | 0.6 |
| GRMZM2G084570-T01 | ZmICK6.1 | 53 (5) RRKK | [K/R]4 | 0.7 |
| GRMZM5G854731-T01 | ZmICK6.2 | 53 (5) RRKK | [K/R]4 | 0.7 |
| GRMZM2G343769-T01 | ZmICK7 | 12 (5) KRKR | [K/R]4 | 0.7 |

## Supplementary Figures

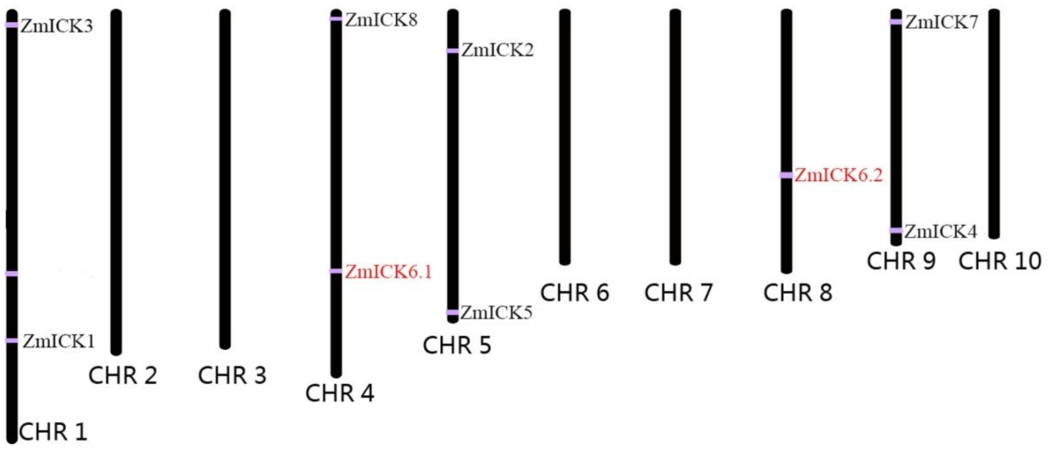


**Figure S1** Physical locations information about maize ICK genes, the grey boxes on chromosomes indicate the approximate locations of the 9 maize ICK genes. CHR is the abbreviation of chromosomes and represents as black bars. The number corresponds to the number of chromosomes.

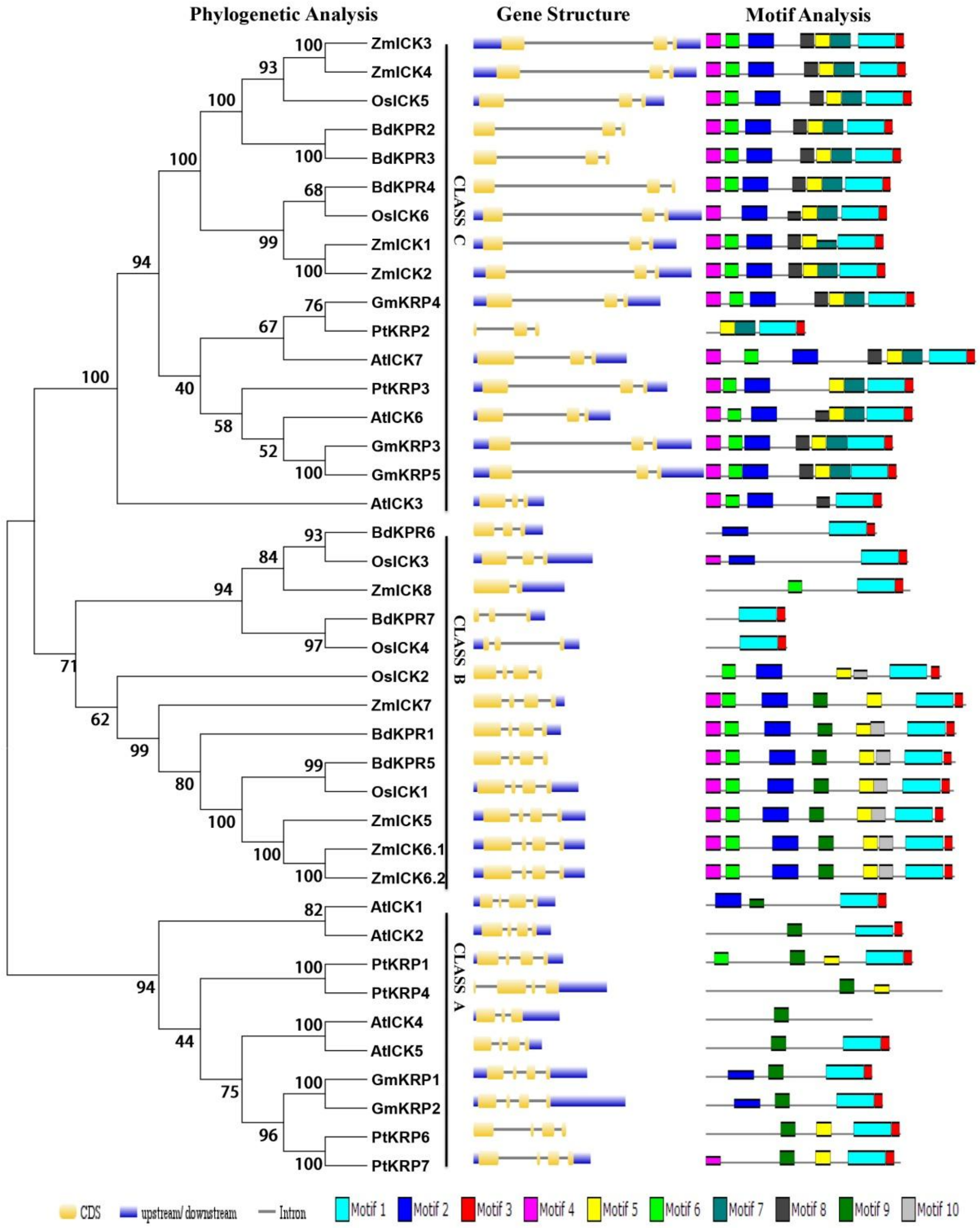


**Figure S2** The primary sequence analysis of 40 ICK/KRP genes from *Arabidopsis thaliana* (*AtICK*), *Oryza sativa* (*OsICK*), *Brachypodium distachyon* (*BdKRP*), *Glycine max* (*GmKRP*), *Populus tric*hocarpa (PtKRP) and Zea mays (ZmICK) contains phylogenetic analysis, gene structure and motif analysis. The different color boxes represent the different motif in the motif analysis. The three main components in gene structure analysis represent CDS region, upstream/downstream and Intron, respectively.

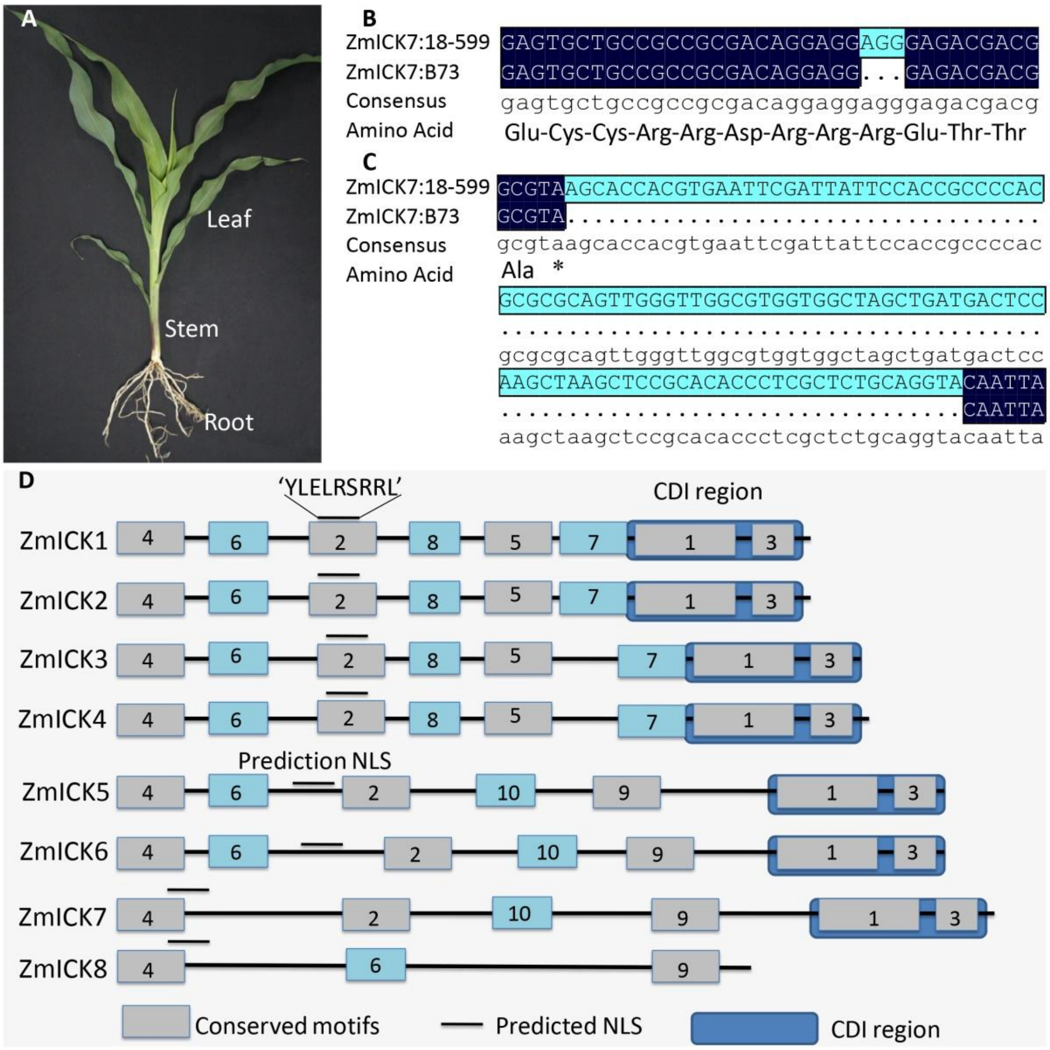


**Figure S3** The results from the analysis of the cloned ZmICK genes. (A) The roots, stems and leaves were collected when maize bred line 18-599 was in the initial jointing stage of growth. (B) and (C) are differences between the cloned sequences from maize bred line 18-599 and the reference sequence of B73. ZmICK7 has a three base pair insertion and led to a single amino acid inserted. ZmICK8 contains a 109bp inserted fragment led to an early termination of translation and lost the conserved CDI region. (D) Conserved motif analysis of the cloned ZmICK proteins sequence. Different coloured boxes represent the conserved motifs, the length of the long black lines represents the amino acid number of the ICK protein sequences, the short black line above the boxes or the long black line represents the predicted nuclear localization signals, and the blue boxes indicate the conserved domain CDI regions.

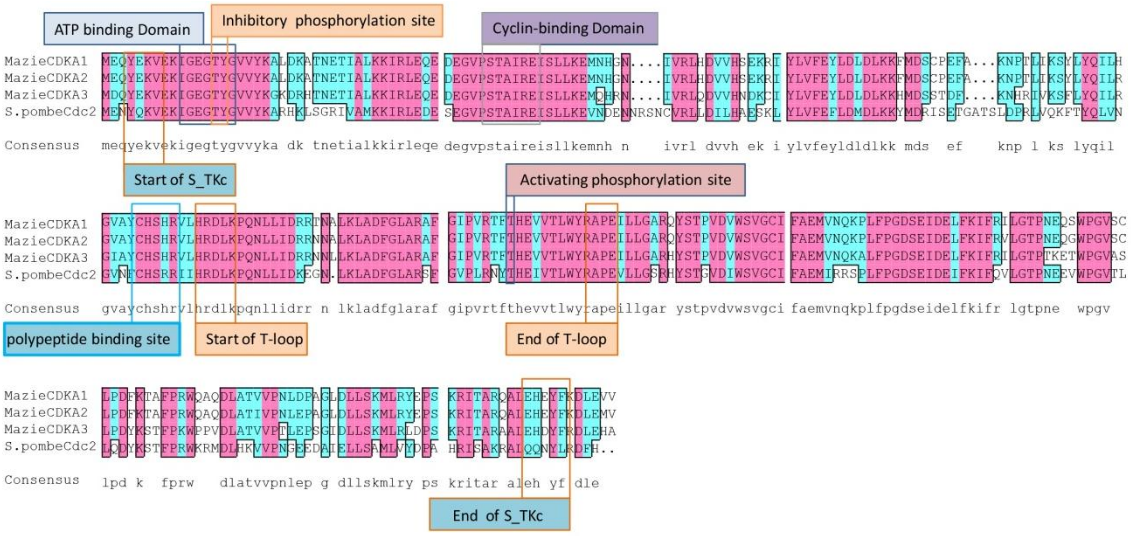


**Figure S4** The protein sequence alignment among three A-type CDKs of maize and Cdc2 of *Schizosaccharomyces pombe*. The specific amino acid sites are marked on the sequence alignment including the conserved S-TKc domain, ATP binding site, Cyclin-binding site and T-loop region.

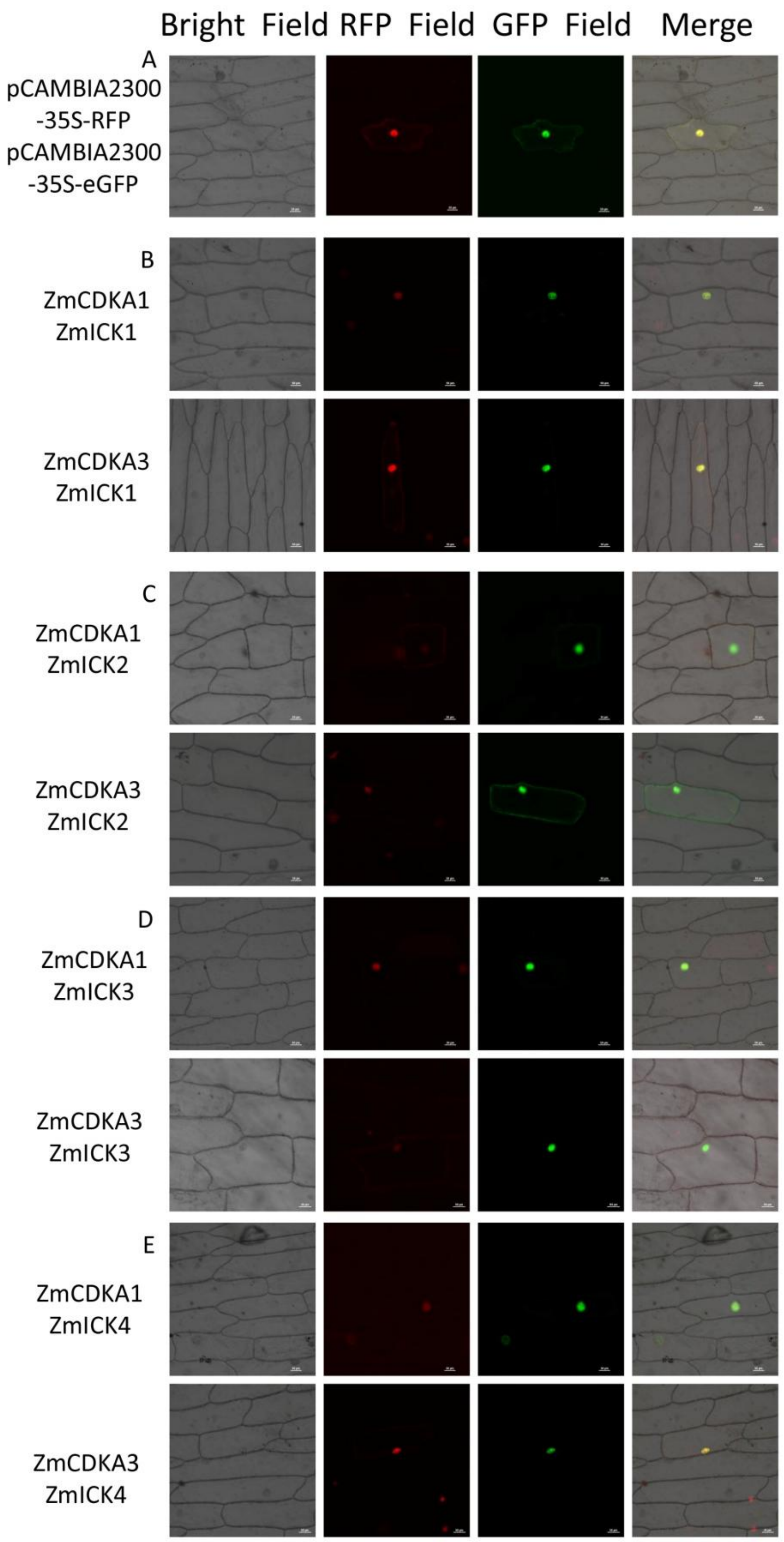


**Figure S5** Co-localization of C-group ZmICKs and A-type CDKs. Combinations of the ZmICK-eGFP fusion protein vectors and the CDKA-RFP fusion protein vectors were transformed into onion epidermal cell with a gene gun and visualized with an ECLIPSE 80i fluorescence microscope. A is the co-localization about control pCAMBIA2300-35S-eGFP

and pCAMBIA2300-35S-RFP. B is the co-localization result of fusion protein ZmICK1-eGFP with ZmCDKA-RFP. C shows the co-localization result of fusion protein ZmICK2-eGFP with ZmCDKA-RFP. D indicates the co-localization result of fusion protein ZmICK3-eGFP with ZmCDKA-RFP. E presents the co-localization result of fusion protein ZmICK4-eGFP with ZmCDKA-RFP. The images were detected under a bright-field, RFP, or GFP view and were then merged three into one. Bar=50 μm.

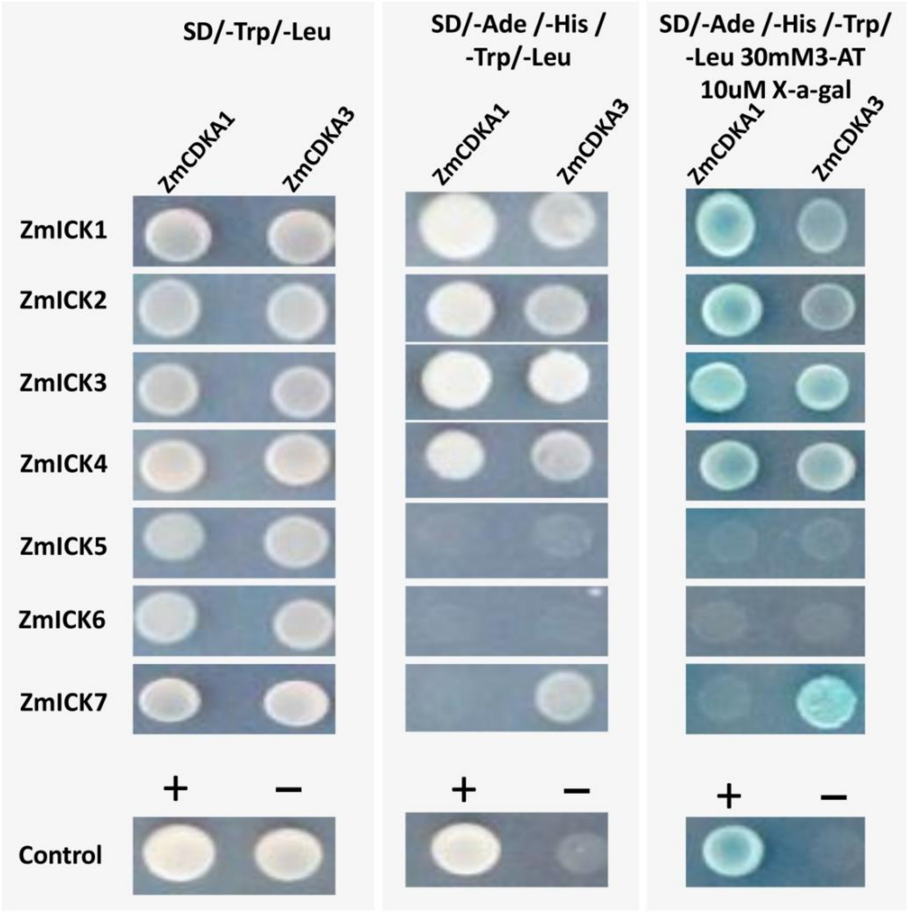


**Figure S6** The results of the yeast two-hybrid analysis of ZmICKs and ZmCDKAs. SD/-Leu/-Trp indicates media lacking tryptophan (Trp) and leucine (Leu). SD/-Ade/-His/-Leu/-Trp represents the synthetic dextrose (SD) media lacking tryptophan (Trp), leucine (Leu), adenine (Ade) and histidine (His). SD/-Ade/-His/-Leu/-Trp/30mM3-AT/10uM X-α-gal indicates the synthetic dextrose (SD) media contain 30 mM 3-AT and 10 uM X-α-gal. The gray box indicates the growth condition on the same medium and every yeast plaque indicates the growth of the transformants. "+" represents the positive control of the yeast two-hybrid, the combination of pGADT7 and pGBKT7-53. "-" shows the negative control of yeast two-hybrid, the pGADT7-T co-transformed with pGBKT7-lam.

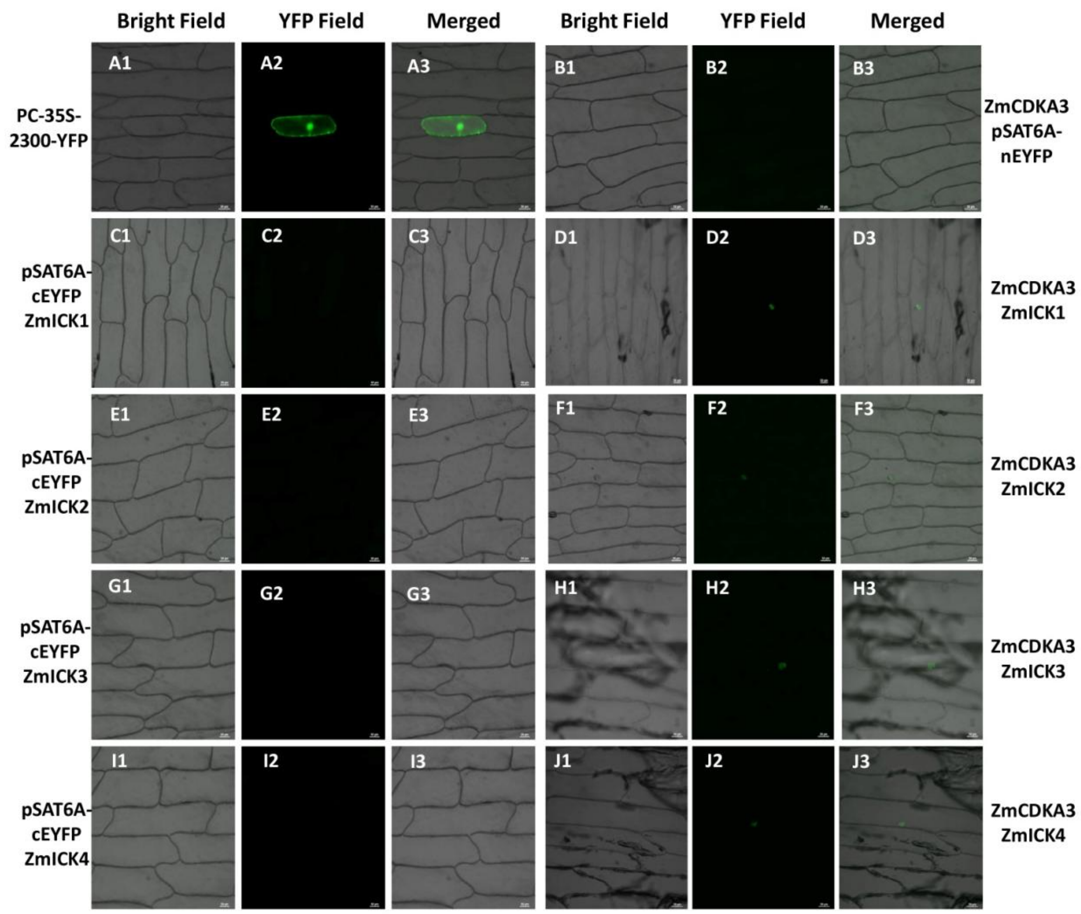


**Figure S7** Bimolecular fluorescence complementation analysis of ZmICK1, ZmICK2, ZmICK3, and ZmICK4 with ZmCDKA3, the pC-35S-2300-eYFP served as a positive control, the pSAT6A-cEYFP and pSAT6-ZmICK-nEYFP, and pSAT6A-ZmCDKA3-cEYFP and pSAT6-nEYFP was used as a negative control. The pSAT6-ZmICK-nEYFP and the corresponding pSAT6-ZmICK-nEYFP and pSAT6A-ZmCDKA3-cEYFP images are shown side-by-side. (A1 - J1) are the detection result under bright-field. (A2-J2) present the detection under the YFP field. (A3-J3) are the merged images of bright field and YFP field. Each alphabet labelled with 1 to 3 represents the same combination. Bar=50 μm.

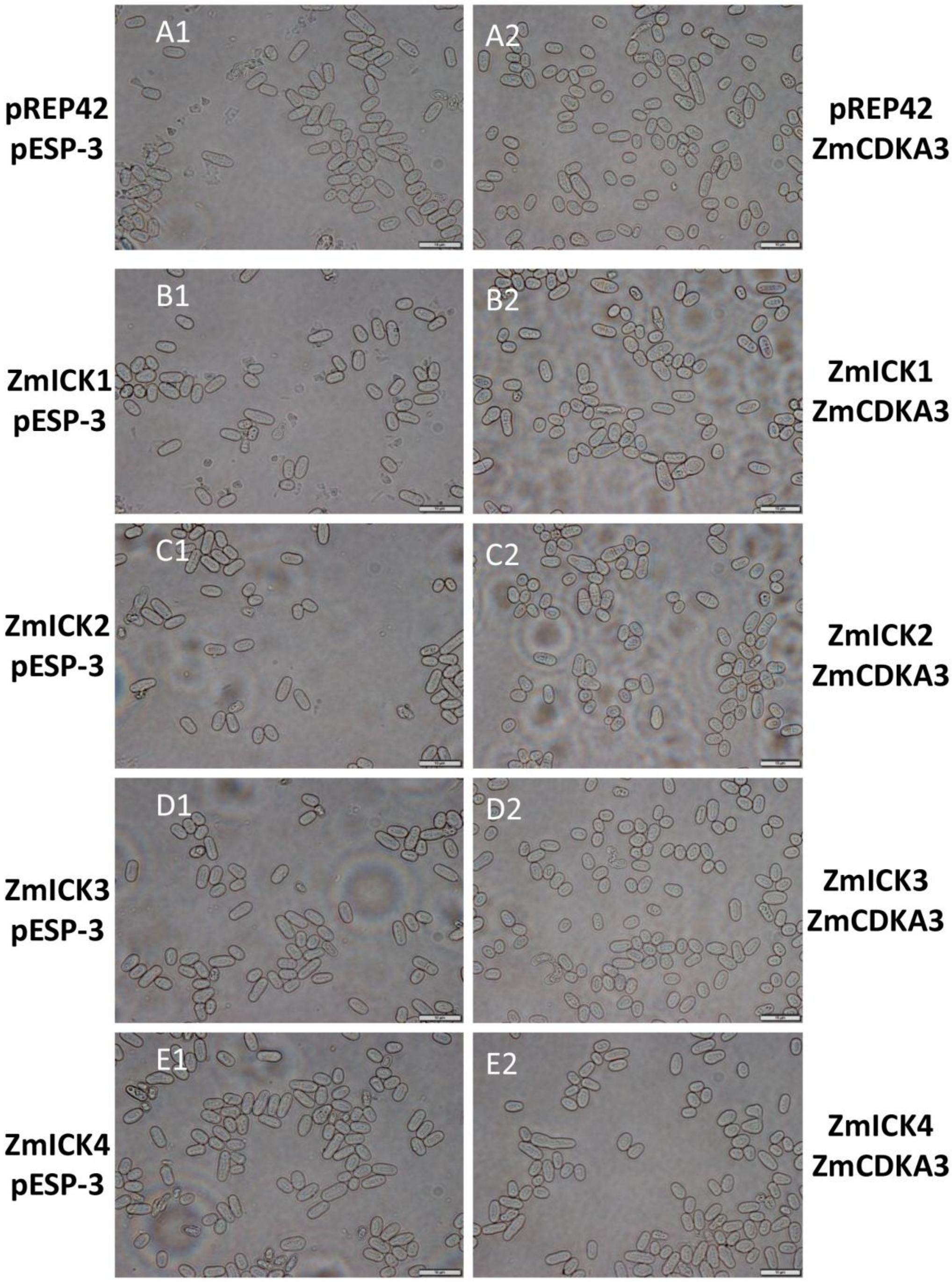


**Figure S8** Cytological analysis of fission yeast cells expressing C-group ZmICKs, ZmCDKA3 and a combination of the proteins. A1 represents wild-type fission yeast cells transformed with pREP42 and pESP-3. A2 shows yeast cells expressing ZmCDKA3 and pREP42. B1 to E1 presents the yeast cells expressing the C-group ZmICKs-pREP42 with pESP-3. B2 to E2 shows the yeast cells co-expressing C-group ZmICKs and ZmCDKA3. The ZmICKs-pREP42 and the corresponding ZmICKs-pREP42 with ZmCDKA3-pESP-3 images are shown side-by-side. Bars=10 μm.

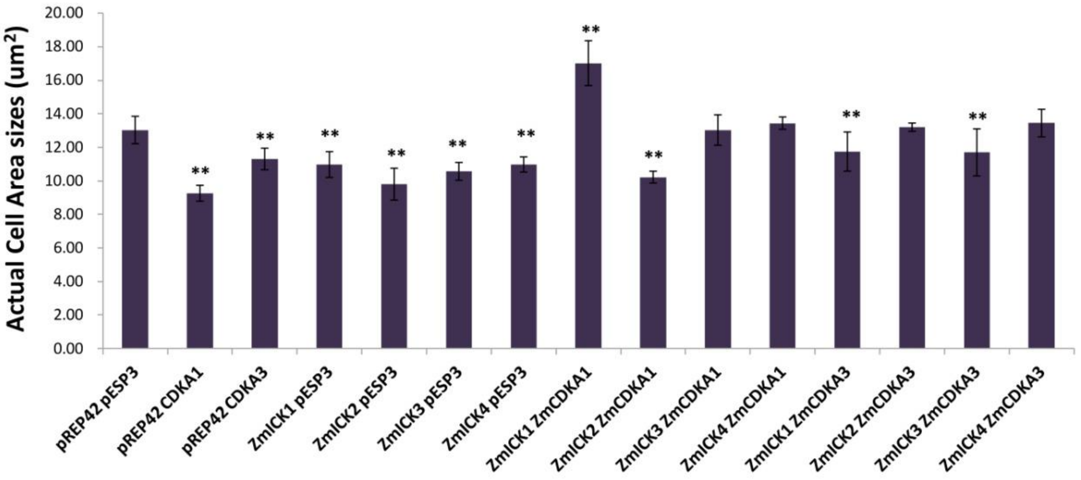


**Figure S9** The averages with standard deviations of the actual cell size ($um^2$). Averages are the average of the transformation of the same combination between different views and standard deviations represent the degree of dispersion of them. The X-axis represents the actual cell area size ($um^2$). The Y-axis shows the different transformation of combination. "**" indicates significant differences in mean values between the different treatments and controls.

```
ZmICK6.1    CTGGGAAAATTATCAGAAGCACCGTACTAGAGAGAGAGAG    40
ZmICK6.2    CTGGGAAAATTATCAGAAGCACCGTACTAGAGAGAGAGAG    40
Consensus   ctgggaaaattatcagaagcaccgtactagagagagagag
ZmICK6.1    AGAGAGAGAATTGCAGCAGAGCTCTTCCGTCCGACAGGCT    80
ZmICK6.2    AGAGAGAGAATTGCAGCAGAGCTCTTCCGTCCGACAGGCT    80
Consensus   agagagagaattgcagcagagctcttccgtccgacaggct
ZmICK6.1    CTCGTCAGTCACAGGCGGATAGAACCCGCCTCCCTGCACT   120
ZmICK6.2    CTCGTCAGTCACAGGCGGATAGAACCCGCCTCCCTGCACT   120
Consensus   ctcgtcagtcacaggcggatagaacccgcctccctgcact
ZmICK6.1    GTTCGCTGGACCGGGCCCGTCCTGCCCCAACCCTGCTGCT   160
ZmICK6.2    GTTCGCTGGACCGGGCCCGTCCTGCCCCAACCCTGCTGCT   160
Consensus   gttcgctggaccgggcccgtcctgccccaaccctgctgct
ZmICK6.1    AAACAGCACGCTTTTTCAGAGAGAAACTTTCAGGAGTTAC   200
ZmICK6.2    AAACAGCACGCTTTTTCAGAGAGAAACTTTCAGGAGTTAC   200
Consensus   aaacagcacgctttttcagagagaaactttcaggagttac
ZmICK6.1    CTATCCTCCTCCACTTCTCTACTGCGTCGCCTGCTCCGGA   240
ZmICK6.2    CTATCCTCCTCCACTTCTCTACTGCGTCGCCTGCTCCGGA   240
Consensus   ctatcctcctccacttctctactgcgtcgcctgctccgga
ZmICK6.1    CCACAACATCCCAGTTTCTTTCGTTCGTCGGGTAATAAGG   280
ZmICK6.2    CCACAACATCCCAGTTTCTTTCGTTCGTCGGGTAATAAGG   280
Consensus   ccacaacatcccagtttctttcgttcgtcgggtaataagg
ZmICK6.1    CTGCACCTCTGCAACGGAGCCCCTGACGTCCGTAACGTTC   320
ZmICK6.2    CTGCACCTCTGCAACGGAGCCCCTGACGTCCGTAACGTTC   320
Consensus   ctgcacctctgcaacggagcccctgacgtccgtaacgttc
ZmICK6.1    TGTCCACGGGAACAAAGCCTGTAACGCAAGAATCGCAACG   360
ZmICK6.2    TGTCCACGGGAACAAAGCCTGTAACGCAAGAATCGCAACG   360
Consensus   tgtccacgggaacaaagcctgtaacgcaagaatcgcaacg
ZmICK6.1    TCCCTCTCTGCGAACTGTACAGTACGGGGCGCGGCCGGTT   400
ZmICK6.2    TCCCTCTCTGCGAACTGTACAGTACGGGGCGCGGCCGGTT   400
Consensus   tccctctctgcgaactgtacagtacggggcgcggccggtt
ZmICK6.1    AAGCACGCCACCGTTCCGCTCCTCGCCGTTAAGATTTTCG   440
ZmICK6.2    AAGCACGCCACCGTTCCGCTCCTCGCCGTTAAGATTTTCG   440
Consensus   aagcacgccaccgttccgctcctcgccgttaagattttcg
ZmICK6.1    AATTCGAAACCCCTCCTGGGCACGGGCACCCAACGGCATG   480
ZmICK6.2    AATTCGAAACCCCTCCTGGGCACGGGCACCCAACGGCATG   480
Consensus   aattcgaaacccctcctgggcacgggcacccaacggcatg
ZmICK6.1    CCAGCCGTCCGATCCGCCGCACTGCACGTGTCAGGCTGAG   520
ZmICK6.2    CCAGCCGTCCGATCCGCCGCACTGCACGTGTCAGGCTGAG   520
Consensus   ccagccgtccgatccgccgcactgcacgtgtcaggctgag
ZmICK6.1    ACCCGCAGGGGCTGTCTGGTAAAAATAATTACGCGTGCCG   560
ZmICK6.2    ACCCGCAGGGGCTGTCTGGTAAAAATAATTACGCGTGCCG   560
Consensus   acccgcaggggctgtctggtaaaaataattacgcgtgccg
ZmICK6.1    TGTGCCAGGCGTCGAAGCTTCCCCCGCTACATTCCGTTTG   600
ZmICK6.2    TGTGCCAGGCGTCGAAGCTTCCCCCGCTACATTCCGTTTG   600
Consensus   tgtgccaggcgtcgaagcttcccccgctacattccgtttg
ZmICK6.1    TGGGCTGCACCTGCACTGCACGCCGTTCCCACCCCCGTGT   640
ZmICK6.2    TGGGCTGCACCTGCACTGCACGCCGTTCCCACCCCCGTGT   640
Consensus   tgggctgcacctgcactgcacgccgttcccacccccgtgt
ZmICK6.1    GCCAGGCGCCGAGGCCGAGCGTACCGGCCGCGGTAGGTGG   680
ZmICK6.2    GCCAGGCGCCGAGGCCGAGCGTACCGGCCGCGGTAGGTGG   680
Consensus   gccaggcgccgaggccgagcgtaccggccgcggtaggtgg
ZmICK6.1    GGCCGGCCGCACGCGACCAGCGCCGTGCGGGCGCTCCCCC   720
ZmICK6.2    GGCCGGCCGCACGCGACCAGCGCCGTGCGGGCGCTCCCCC   720
Consensus   ggccggccgcacgcgaccagcgccgtgcgggcgctccccc
ZmICK6.1    AACGGACAGATCCGGCTAACGTCGCCGTCAGGGAAGGGCC   760
ZmICK6.2    AACGGACAGATCCGGCTAACGTCGCCGTCAGGGAAGGGCC   760
Consensus   aacggacagatccggctaacgtcgccgtcagggaagggcc
ZmICK6.1    GATCCTGGCCGTCGGATCCGGATCCATTCCCCTCGGCCGC   800
ZmICK6.2    GATCCTGGCCGTCGGATCCGGATCCATTCCCCTCGGCCGC   800
Consensus   gatcctggccgtcggatccggatccattcccctcggccgc
ZmICK6.1    GGCATCTTATATTTGTAGGCGCACCCGAGCGCCCGACGGG   840
ZmICK6.2    GGCATCTTATATTTGTAGGCGCACCCGAGCGCCCGACGGG   840
Consensus   ggcatcttatatttgtaggcgcacccgagcgcccgacggg
ZmICK6.1    TGCGTGATTGTTGTAGTAGAATCCAAAGCGCAAGCGGCTG   880
ZmICK6.2    TGCGTGATTGTTGTAGTAGAATCCAAAGCGCAAGCGGCTG   880
Consensus   tgcgtgattgttgtagtagaatccaaagcgcaagcggctg
ZmICK6.1    CAGCCTGCAGGCAGCGCCGCGCAGGCGTGGGAGTGGCCGA   920
ZmICK6.2    CAGCCTGCAGGCAGCGCCGCGCAGGCGTGGGAGTGGCCGA   920
Consensus   cagcctgcaggcagcgccgcgcaggcgtgggagtggccga
ZmICK6.1    GTGGGAGTGGGAGTGAAAAAGAGGAACCGGCCAAGAGAAG   960
ZmICK6.2    GTGGGAGTGGGAGTGAAAAAGAGGAACCGGCCAAGAGAAG   960
Consensus   gtgggagtgggagtgaaaaagaggaaccggccaagagaag
ZmICK6.1    CAAGCGAGAAGAAGGCAGTGCTGCGGCGGCGTTCCGTAA    999
ZmICK6.2    CAAGCGAGAAGAAGGCAGTGCTGCGGCGGCGTTCCGTAA    999
Consensus   caagcgagaagaaggcagtgctgcggcggcgttccgtaa
```

**Figure S10** The 1000bp upstream sequence alignment between ZmICK6.1 and ZmICK6.2
